# RAB4A acts as a negative feedback regulator of extracellular vesicle secretion during TGF‐β signaling

**DOI:** 10.1111/febs.70520

**Published:** 2026-03-30

**Authors:** Dorival Mendes Rodrigues‐Junior, Maria Anastasia Doumani, Hao Fu, Jelle van den Bor, Olof Idevall‐Hagren, Aristidis Moustakas

**Affiliations:** ^1^ Department of Medical Biochemistry and Microbiology, Science for Life Laboratory, Box 582, Biomedical Center Uppsala University Sweden; ^2^ Department of Biochemistry University of Yamanashi Japan; ^3^ Department of Cell Biology, Neurobiology and Biophysics Utrecht University The Netherlands; ^4^ Department of Medical Cell Biology Uppsala University Sweden

**Keywords:** cancer, ESCRT, extracellular vesicles, Rab4A, tetraspanin, TGF‐β

## Abstract

Extracellular vesicles (EVs) secreted by cancer cells actively modulate the tumor microenvironment, thereby promoting cancer progression. Transforming growth factor‐β (TGF‐β) signaling has been implicated in the regulation of EV biogenesis, yet the molecular mechanisms underlying this process have only recently begun to emerge. In this study, we investigated TGF‐β‐responsive mediators that regulate EV release in lung, breast, and ovarian carcinoma cells by modulating the expression and activity of genes associated with EV biogenesis, including components of the ESCRT machinery, tetraspanins, and Rab GTPases. We found that TGF‐β selectively enhances the mRNA expression of *PDCD6IP* (*ALIX*), *CD81*, *ARF6*, and *RAB4A* in a cell type–specific manner. R‐SMAD silencing had clear negative effects on the regulation of *ALIX* or *RAB4A*, whereas AKT kinase inhibition suppressed the induction of *ALIX* and *CD81*. Additionally, TGF‐β stimulation increased ALIX S‐palmitoylation, consistent with enhanced ALIX–TSG101 complex formation on vesicular membranes. However, knockdown of ALIX or CD81 did not impair TGF‐β‐induced EV secretion. On the contrary, TGF‐β‐induced upregulation of RAB4A expression is functionally unique because RAB4A facilitates fast endosomal recycling, a process that limits EV release. Accordingly, silencing RAB4A significantly increased the fusion of multivesicular bodies with the plasma membrane followed by EV secretion, suggesting that TGF‐β‐induced RAB4A acts as a negative feedback regulator of EV release. Our findings reveal a novel mechanism by which RAB4A modulates TGF‐β‐driven EV production by cancer cells.

## Graphical abstract

TGF‐β signaling regulates extracellular vesicle (EV) release in cancer cells by modulating the expression and activity of genes associated with EV biogenesis. The TGF‐β‐induced upregulation of RAB4A expression facilitates fast endosomal recycling, a process that limits the fusion of multivesicular bodies with the plasma membrane and EV secretion. Hence, this study suggests that TGF‐β‐induced RAB4A acts as a negative feedback regulator of TGF‐β‐driven EV production by cancer cells.

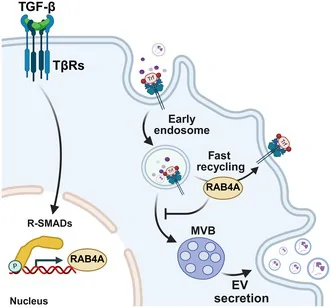

**Abbreviations**
Acyl‐RAC: Acyl‐resin‐assisted capture
AKTi: AKT inhibitor
BRCA: breast cancer
COAD: colon adenocarcinoma
DMSO: dimethyl‐sulfoxide
EEA1: early endosome antigen 1
EMT: epithelial to mesenchymal transition
ESCRT: endosomal sorting complexes required for transport
EV: extracellular vesicle
GBM: glioblastoma
GOBP: gene ontology for biological process
GSEA: gene set enrichment analysis
Hi‐C: high‐throughput chromosome conformation capture
HNSCC: head neck squamous cell carcinoma
LIHC/HCC: liver hepatocellular carcinoma
LUAD: lung adenocarcinoma
MEKi: MEK inhibitor
MVB: multivesicular body
NTA: nanoparticle tracking analysis
OV: ovarian serous cystadenocarcinoma
PAAD: pancreatic adenocarcinoma
STRING: search tool for the Retrieval of Interacting Genes
TAD: transcriptional activation domains
TCGA: The Cancer Genome Atlas
TEM: transmission electron microscopy
TF: transcription factor
TGF‐β: transforming growth factor‐β
TGFβR1: TGF‐β receptor type 1
TGFβR2: TGF‐β receptor type 2
TIRF: total internal reflection fluorescence
TNBC: triple negative breast cancer
Trf: transferrin
TSS: transcription start site
VSF: vesicular secretome fraction

## Introduction

The mechanisms regulating the biogenesis of tumor‐derived extracellular vesicles (EVs) are of critical importance to elucidate the processes underlying tumorigenesis [1]. These lipid bilayer‐enclosed nanoparticles carry bioactive molecules, including proteins, nucleic acids, lipids, and carbohydrates, mediating the communication between cancer cells and cells in their surrounding microenvironment or even at distant tissue locations [2]. EV‐mediated intercellular communication is essential for driving key hallmarks of tumor progression, including the formation of premetastatic niches at distant sites [1]. Among the various signaling molecules associated with EVs, transforming growth factor‐β (TGF‐β) has been implicated in promoting epithelial to mesenchymal transition (EMT), immune suppression, maintenance of stem cell populations, and therapeutic resistance of tumor cells, as well as influencing EV biogenesis [3, 4]. However, the molecular mechanisms controlling the TGF‐β regulatory role on EV biogenesis have only recently begun to be understood [4, 5].

TGF‐β and its related family members signal through serine/threonine kinase receptors, specifically type 1 (TGFβR1) and type 2 (TGFβR2) receptors, which phosphorylate SMAD proteins (SMAD2 and SMAD3) and activate in parallel non‐SMAD pathways, inducing MAP‐kinases and phosphatidylinositol 3‐kinase/AKT [6]. This molecular cascade mediates cytoplasmic signaling and regulates the transcription of several target genes [3]. Our recent findings demonstrated that TGF‐β increases the secretion of tumor‐derived EVs via sustained MEK/ERK1/2 signaling, with cholesterol biosynthesis being a key intermediate step in this process [7]. Furthermore, TGF‐β can enhance EV release from cancer‐associated fibroblasts [8] and from nonsenescent fibroblasts in other pathological contexts [9]. On the contrary, TGF‐β1 stimulation did not affect the release of EVs from human pulmonary artery smooth muscle cells that are implicated in arterial hypertension [10], and experimental conditions have been reported under which TGF‐β may even suppress EV release [11]. These limited cases indicate that the mechanism driven by TGF‐β may be diverse and complex. Thus, the specific mechanisms by which TGF‐β can modulate tumor‐derived EVs to assist tumorigenesis remain open to deeper understanding.

The biogenesis of small EVs (≤ 200 nm) is a multistep process tightly regulated by extracellular stimuli. For instance, these stimuli can activate intracellular signaling pathways, for example, MEK/ERK1/2 kinases [12], which in turn activate downstream effectors. The best‐established effectors are the endosomal sorting complexes required for transport (ESCRT) machinery, tetraspanins, lipid/cholesterol metabolic processes or Rab GTPases that control endosomal trafficking [2]. These components work in a coordinated manner to boost the secretion of tumor‐derived EVs, including either exosomes, originating from multivesicular bodies (MVBs) of the endosomal system, or microvesicles that originate from the plasma membrane [2]. Here, we investigated whether TGF‐β signaling, which engages cholesterol biosynthesis, regulates additional molecular components mediating the secretion of tumor‐derived EVs. Interestingly, we found that TGF‐β enhances RAB4A expression, which mediates short‐loop feedback and restricts TGF‐β‐induced EV secretion.

## Results

### TGF‐β signaling modulates the expression of genes regulating EV biogenesis

**Fig. 1:**
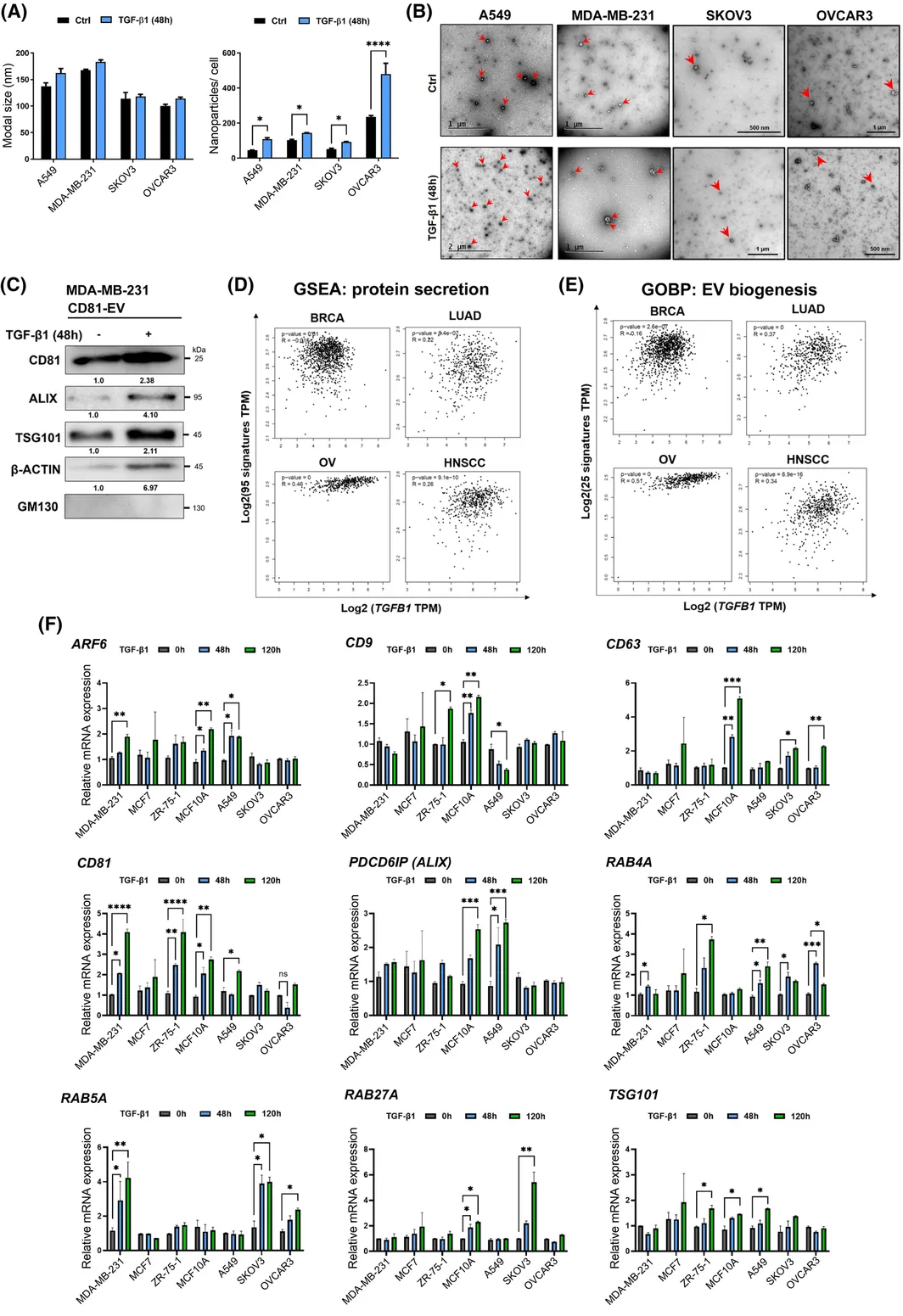
TGF‐β regulates the expression of genes involved in extracellular vesicle (EV) biogenesis. (A) EVs released by A549, MDA‐MB‐231, SKOV3, and OVCAR3 cells were quantified by nanoparticle tracking analysis (NTA) in terms of particle modal size (left) and particle number after normalization to the total cell number (right). The cells were stimulated with vehicle (Ctrl) or 5 ng·mL^−1^ TGF‐β1 for 48 h. (B) Representative transmission electron microscopy (TEM) pictures of EVs isolated as VSF from A549, MDA‐MB‐231, SKOV3, and OVCAR3 cells stimulated or not (−) with 5 ng·mL^−1^ TGF‐β1 for 48 h. Scale bars 500 nm, 1 or 2 μm, accordingly indicated in the figure. Red arrows mark specific EVs. (C) Protein expression levels of the indicated EV‐specific protein markers in EV extracts (isolated after CD81‐specific enrichment) derived from MDA‐MB‐231 cells stimulated or not (−) with 5 ng·mL^−1^ TGF‐β1 for 48 h. (D, E) Pearson correlation analysis of mRNA expression in BRCA, LUAD, OV, and HNSCC of gene signatures for protein secretion (D) and extracellular vesicle biogenesis (E) relative to the *TGFB1* mRNA expression, measured as transcripts per million (TPM) transformed by log_2_; data were obtained from The Cancer Genome Atlas (TCGA). *P*‐ and *R*‐values are listed. (F) RT‐qPCR analysis of the indicated mRNA levels in MDA‐MB‐231, MCF7, ZR‐75‐1, MCF10A, A549, SKOV3, and OVCAR3 cells upon stimulation with 5 ng·mL^−1^ TGF‐β1 for 48 or 120 h. Values represent fold‐change of mRNA expression normalized to *GAPDH* and expressed relative to the level at 0 h TGF‐β1 (Ctrl). Data in A and F are presented as mean values of three biological replicates ± SEM, each in technical duplicates and *P*‐values are shown based on two‐way ANOVA, followed by multiple paired comparisons conducted by means of Bonferroni's post‐test method. *P*‐values: *P ≤ 0.05; **P ≤ 0.01; ***P ≤ 0.001; ****P ≤ 0.0001.

We corroborated our previous findings [7] by demonstrating that TGF‐β1 stimulation significantly enhances the secretion of tumor‐derived EVs from A549 (lung adenocarcinoma; LUAD), MDA‐MB‐231 (triple negative breast cancer; TNBC), SKOV3 and OVCAR3 (ovarian serous cystadenocarcinoma; OV) cells, without altering their modal nanoparticle size, as assessed by nanoparticle tracking analysis (NTA) (Figs 1A, S1A). Transmission electron microscopy (TEM) confirmed the presence of membrane‐enclosed nanoparticles enriched in the vesicular secretome fraction (VSF) isolated from the cell‐ and serum‐free media (Fig. 1B). The tetraspanin CD81‐positive EV subpopulation was enriched from an equal volume of MDA‐MB‐231 VSF and was characterized by the presence of EV markers (CD81, ALIX, TSG101, and β‐ACTIN) and the absence of the Golgi marker GM130. The increased levels of these EV markers following TGF‐β stimulation indicate a higher number of EVs within the same preparation volume (Fig. 1C), supporting our NTA data.

Notably, among the three TGF‐β family members, TGF‐β1 has been more widely linked to different manifestations of cancer development, ranging from the early onset of carcinogenesis to advanced stages of disease, such as metastasis and resistance to treatment with diverse medications, including immunotherapy [3, 13]. Hence, in addition to cholesterol biosynthesis [7], we sought novel mediators of EV biogenesis that could potentially be regulated by TGF‐β signaling. Thus, The Cancer Genome Atlas (TCGA) patient tumor datasets were used to link *TGFB1* mRNA expression with the signature of genes related to pathways that could mediate EV biogenesis. Primarily, Gene Set Enrichment Analysis (GSEA) of tumor datasets from patients with breast cancer (BRCA), LUAD, OV, head neck squamous cell carcinoma (HNSCC), colon adenocarcinoma (COAD), glioblastoma (GBM), liver hepatocellular carcinoma (LIHC or HCC), and pancreatic adenocarcinoma (PAAD) revealed positive and significant correlations between *TGFB1* mRNA expression and the signature of genes classified as hallmarks for protein secretion (Figs 1D, S1B and Table S1, note the lack of significance in BRCA). Additionally, *TGFB1* mRNA expression was positively and significantly correlated with the gene ontology for biological process (GOBP) associated with EV biogenesis across multiple cancer type datasets (Figs 1E, S1C and Table S1). Even though various tumor types exhibited different correlation patterns among the genes associated with the protein secretion hallmark and EV biogenesis, it is important to mention that mRNA expression correlations cannot demonstrate causative relationships. Hence, we investigated whether TGF‐β could functionally regulate the mRNA expression of genes established as regulators of vesicular trafficking and EV biogenesis, such as *ARF6*, the ESCRT components *TSG101* and *PDCD6IP* (*ALIX*), the tetraspanins *CD9*, *CD63*, and *CD81*, and the RAB GTPases *RAB4A*, *RAB5A*, and *RAB27A* [14], in a panel of breast epithelial cells (MDA‐MB‐231: TNBC; MCF7: luminal BRCA; and MCF10A: non‐tumorigenic), LUAD cells (A549), and OV cells (SKOV3 and OVCAR3) (Figs 1F, S1D).

Of note, TGF‐β1 stimulation for 48 or 120 h induced the expression of *SERPINE1* (i.e., plasminogen activator inhibitor 1; PAI1), used as a positive control for TGF‐β signaling activation in all cell lines (Fig. S1E). *ARF6* mRNA expression was increased significantly in MDA‐MB‐231, MCF10A, and A549 cells. Tetraspanin *CD9* and *CD63* mRNA expression was significantly increased in the non‐tumorigenic MCF10A cells; *CD9* mRNA was additionally enhanced in ZR‐75‐1, while *CD63* was increased in the OV cells. *CD81* mRNA was enhanced significantly by TGF‐β activation in several cell lines, but not in MCF7 or OV cells. *PDCD6IP* (*ALIX*) mRNA levels were elevated only in MCF10A and A549 cells. *RAB4A* mRNA levels were enhanced significantly by TGF‐β activation in several cells, but not in MCF7 or MCF10A cells, whereas *RAB5A* mRNA levels were significantly increased after TGF‐β1 stimulation in MDA‐MB‐231 and in the OV cells, and *RAB27A* mRNA was induced significantly only in MCF10A and in SKOV3 cells. Finally, the mRNA expression of *TSG101* was increased significantly in ZR‐75‐1 and MCF10A cells. Furthermore, focusing on the LUAD model A549, indicated that the expression of *ARF6*, *CD81*, *ALIX*, *RAB4A*, and *TSG101* were induced by TGF‐β1 with cell type–specific kinetics (Fig. 1F). Examining the early signaling effects of TGF‐β stimulation in A549 cells revealed a significant increase in expression only for *ALIX* mRNA (Fig. S1F).

The mRNA expression analyses revealed that TGF‐β signaling does not exhibit robust or universal effects on the expression of *ARF6*, ESCRT family, tetraspanin family, and RAB family of genes (Figs 1F, S1F). This suggests cell type–specific differences in the time‐dependent patterns of gene induction, which did not correlate with the basal expression levels of the tested genes in each cell line (Fig. S1D).

### TGF‐β receptor‐SMAD signaling regulates *CD81* , *ALIX* , and *RAB4A* mRNA expression

**Fig. 2:**
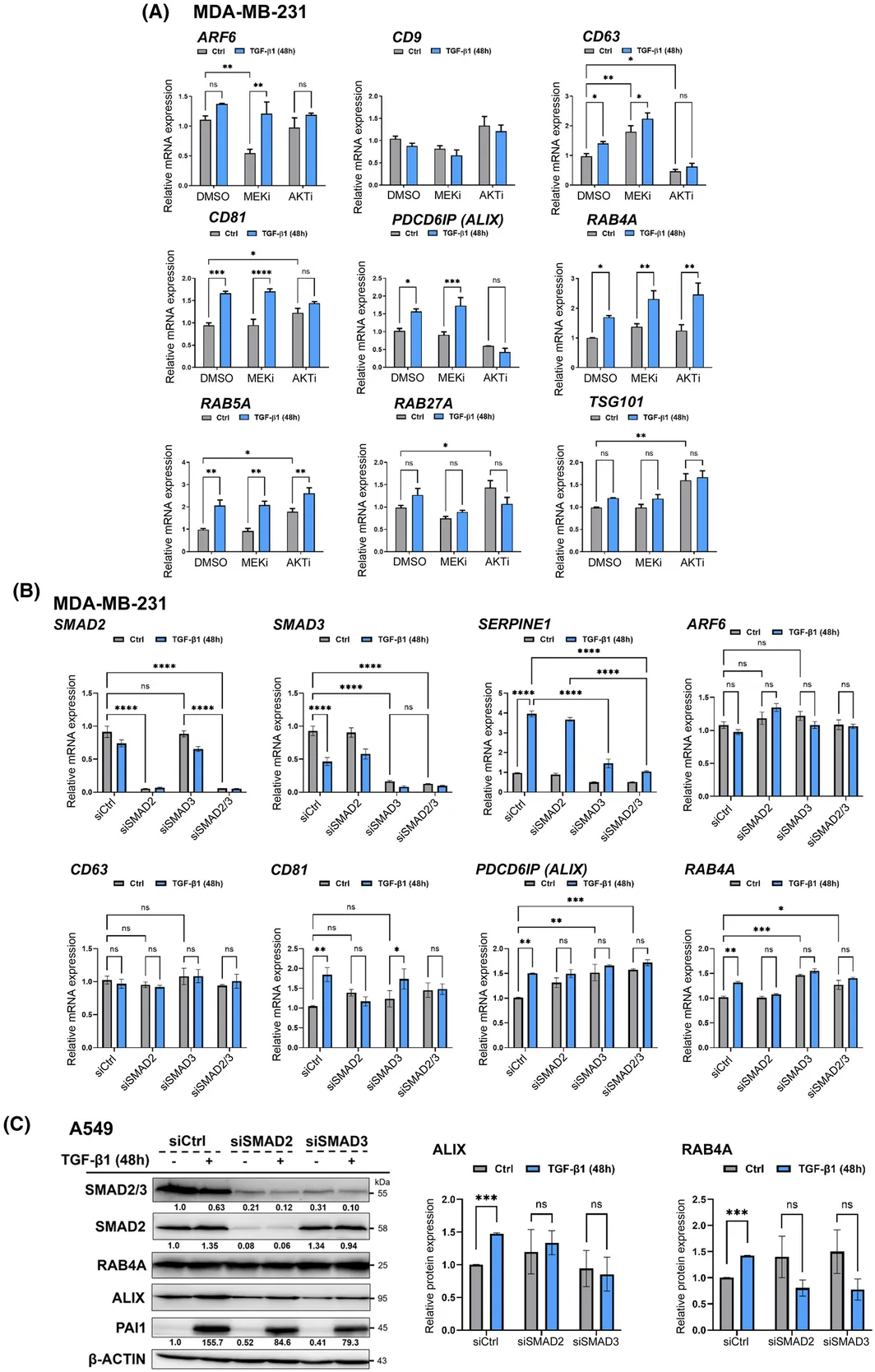
TGF‐β‐induced *CD81*, *ALIX*, and *RAB4A* mRNA expression requires R‐SMAD signaling. (A) RT‐qPCR analysis of the indicated mRNA levels in MDA‐MB‐231 cells stimulated with vehicle (Ctrl) or 5 ng·mL^−1^ TGF‐β1 for 48 h in the absence [dimethyl‐sulfoxide (DMSO) vehicle, Ctrl] or presence of 5 μm MEKi (U0126) or 1.25 μm AKTi (Capivasertib). (B) RT‐qPCR analysis of the indicated mRNAs in MDA‐MB‐231 cells after transient transfection with siCtrl, SMAD2‐specific, SMAD3‐specific siRNAs or their combination after stimulation of the transfected cells with 5 ng·mL^−1^ TGF‐β1 for 48 h. Values represent fold change of mRNA expression normalized to *GAPDH* and expressed relative to the level at siCtrl. (C) Protein expression levels of SMAD2/3, SMAD2, RAB4A, ALIX, PAI1, and β‐Actin (as loading control) in A549 protein extracts transiently transfected with the indicated siRNAs. Densitometric values of three biological replicates were normalized to the control siRNA. The data in A–C are presented as mean values of at least two biological replicates ± SEM, in technical triplicates (A, B) and *P*‐values are shown based on two‐way ANOVA, followed by multiple paired comparisons conducted by means of Tukey's post‐test method: *P*‐values: *P ≤ 0.05; **P ≤ 0.01; ***P ≤ 0.001; ****P ≤ 0.0001; ns, not significant.

Previously, we showed that TGF‐β signaling promotes EV secretion via MEK/ERK1/2‐dependent mechanism [7]. To further investigate whether the above EV biogenesis‐related genes are regulated by MEK/ERK1/2 signaling, we analyzed their mRNA levels in MDA‐MB‐231 cells treated with a non‐toxic concentration (5 μm) of the MEK inhibitor U0126 (MEKi) (Fig. S2A), in the presence or absence of TGF‐β1 for 48 h (Fig. 2A). Unexpectedly, *CD63* mRNA expression was significantly enhanced when MEK signaling was blocked. The mRNA levels of *CD9*, *RAB27A*, and *TSG101* remained unchanged upon MEK inhibition (Fig. 2A), which is consistent with our earlier data that these genes are not regulated by TGF‐β in MDA‐MB‐231 cells (Fig. 1F). Furthermore, the mRNA expression of *SERPINE1*, *CD81*, *ALIX*, *RAB4A*, and *RAB5A* was not significantly altered by MEKi in the context of TGF‐β stimulation (Figs 2A, S2B). These findings indicate that the transcriptional regulation of the above genes is not causatively mediated by TGF‐β‐induced MEK/ERK signaling.

In addition to MEK/ERK signaling, TGF‐β promotes PI3K‐AKT signaling in cancer cells to boost cell migration [15]. Thus, to investigate the impact of AKT signaling regulating the mRNA expression of the EV biogenesis‐related genes, MDA‐MB‐231 cells were treated with 1.25 μm of the AKT inhibitor (AKTi; Capivasertib) [16] (Fig. S2A) in the presence or absence of TGF‐β1 stimulation for 48 h (Fig. 2A). AKTi treatment alone led to a significant increase in *CD81*, *RAB5A*, *RAB27A*, and *TSG101* basal mRNA levels and a decrease in CD63 basal mRNA levels. Among the TGF‐β‐regulated genes, blocking PI3K‐AKT signaling significantly inhibited the induction of *CD63*, *CD81*, and *PDCD6IP* (*ALIX*), mRNA expression. In contrast, the expression of *ARF6*, *CD9*, and *RAB4A* remained unaffected by AKTi treatment. These findings suggest that TGF‐β signaling may regulate the expression of *CD63*, *CD81*, and *ALIX* through activation of the PI3K‐AKT pathway.

Another key substrate of the TGF‐β receptor type I is the SMAD family of proteins (i.e., SMAD2 and SMAD3). Upon phosphorylation, these receptor‐regulated SMADs (R‐SMADs) translocate to the nucleus, regulating gene expression at several genomic loci, together with other transcription factors (TFs) and chromatin‐associated proteins [17]. To investigate the role of R‐SMADs modulating the mRNA expression of EV biogenesis‐related genes (Fig. 1), we performed genetic silencing of SMAD2, SMAD3, or both in MDA‐MB‐231, A549, and MCF7 cells (Figs 2B, S2C,D). Interestingly, R‐SMAD silencing in MDA‐MB‐231 cells impaired the TGF‐β‐induced upregulation of *SERPINE1* (used as a positive control), as well as *CD81*, *ALIX*, and *RAB4A* mRNA. On the contrary, the transcript levels of *ARF6* and *CD63*, whose expression was not affected by TGF‐β stimulation for 48 h, remained unchanged upon R‐SMAD depletion in MDA‐MB‐231 cells (Fig. 2B). Likewise, the absence of R‐SMAD in A549 cells, particularly of SMAD2, inhibited the mRNA expression of *ALIX* and *RAB4A* induced by TGF‐β (Fig. S2C). Furthermore, in MCF7 cells, where the transcriptional response of the EV biogenesis‐related genes to TGF‐β was generally weaker, the lack of R‐SMADs had minimal impact (Fig. S2D). Hence, these data pointed out to R‐SMADs as potential modulators of TGF‐β‐induced mRNA expression of *CD81*, *PDCD6IP* (*ALIX*), and *RAB4A*. Furthermore, silencing SMAD2 or SMAD3 individually attenuated TGF‐β‐induced expression of RAB4A, ALIX, and PAI1 proteins in A549 cells (Fig. 2C). Based on this result and the conclusion of the analysis of Fig. 1, we continued our investigation by focusing on three factors, ALIX, CD81, and RAB4A, which represent the three functional groups of EV biogenesis regulation (ESCRT, tetraspanins and RAB GTPases, respectively).

**Fig. 3:**
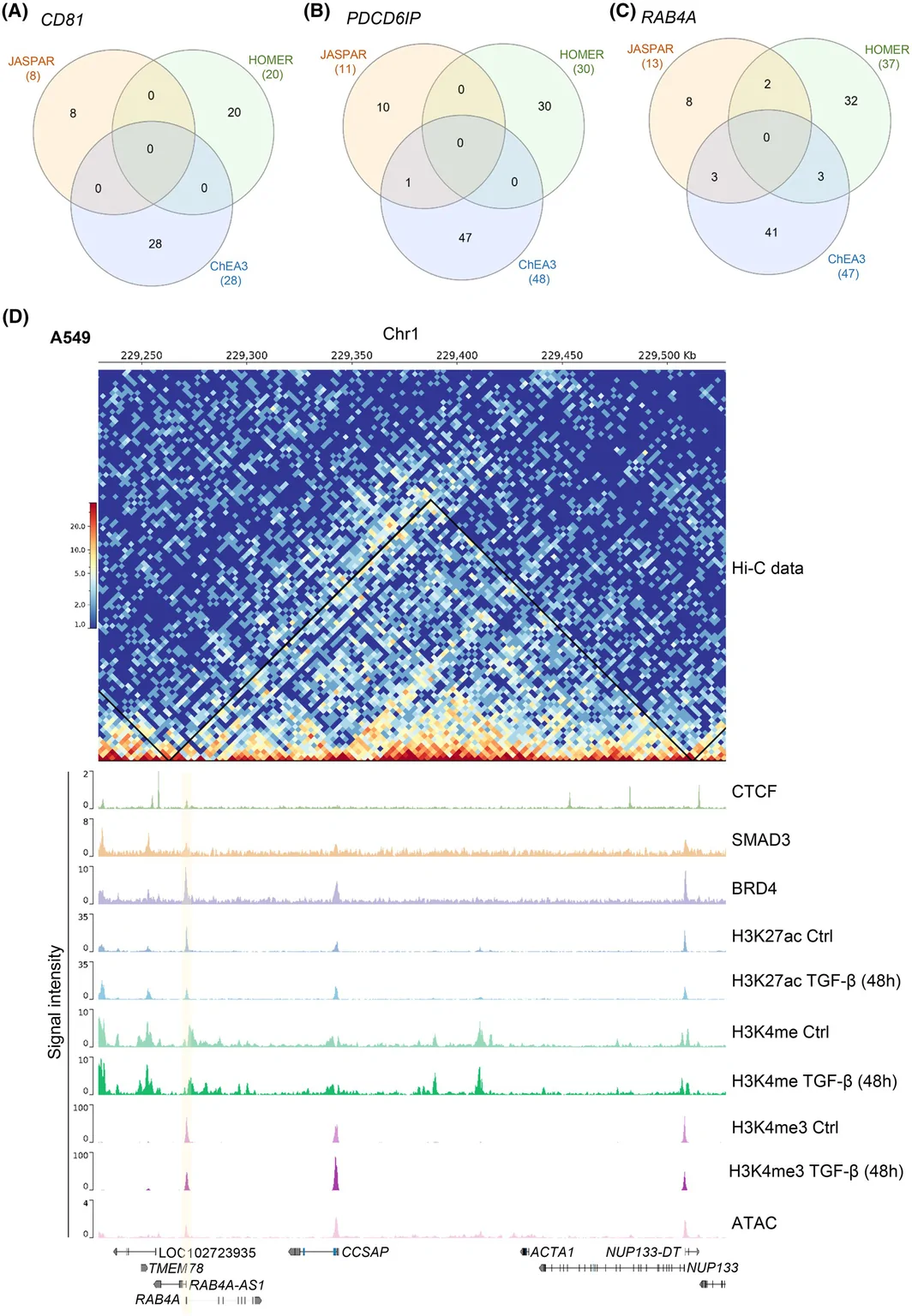
Regulatory landscape of R‐SMAD and associated transcription factors in *CD81*, *ALIX*, and *RAB4A* regions. (A–C) Venn diagram showing the number of significant DNA binding motifs on the *CD81* (A), *PDCD6IP*–ALIX (B), and *RAB4A* (C) promoter sequence using JASPAR, HOMER, and ChEA3 analysis. (D) Genomic landscape of the *RAB4A* locus in A549 cells. High‐throughput chromosome conformation capture (Hi‐C) 1D interaction frequency heatmap in A549 cells (top, 3 kbp resolution). Black triangles in the heatmap indicate topologically associating domains (TADs), which were predicted with a 3 kbp step. CTCF, SMAD3 (TGF‐β‐stimulated for 1.5 h), BRD4‐ChIP‐seq profiles in the genomic region surrounding the *RAB4A* locus in A549 cells (GSE92782, GSE51510, and GSE226487). ChIP‐seq profiles of H3K27ac, H3K4me, and H3K4me3 with or without TGF‐β stimulation for 48 h (CRA001325) are presented.

We investigated potential TFs with predicted binding motifs upstream of the transcription start site (TSS) of *CD81*, *PDCD6IP* (*ALIX*), and *RAB4A* (Fig. 3A–C) by using three independent databases of TF binding profiles (i.e., ChEA3, JASPAR, and HOMER) [18, 19, 20]. Of note, the ChEA 2022 database identified R‐SMADs with binding motifs at the TSS region of *CD81* (among 28 possible TFs), *ALIX* (among 48 possible TFs) and *RAB4A* (among 47 possible TFs) (Tables S2–S4), which is consistent with the R‐SMAD silencing data in MDA‐MB‐231 and A549 cells. This motif enrichment analysis highlights a potential regulatory role for R‐SMADs and raises the possibility that several R‐SMAD cooperating TFs can mediate the TGF‐β‐induced upregulation of *CD81*, *PDCD6IP* (*ALIX*), and *RAB4A* transcripts.

In order to deepen the analysis of R‐SMAD involvement in regulation of these three genes, we re‐analyzed published SMAD3 ChIP‐seq data from A549 cells stimulated with TGF‐β (GSE51510) [21] and identified experimentally verified SMAD3‐binding sites in the regulatory sequences of these genes (Fig. 3D and Figs S3, S4). This dataset also includes high‐throughput chromosome conformation capture (Hi‐C) analysis and thus provided us with information on transcriptional activation domains (TAD) in the loci covering these three genes (Fig. 3D and Figs S3, S4). The *RAB4A* TSS presented a weak but significant binding of SMAD3 (Fig. 3D). This region also bound the chromatin regulator BRD4, based on BRD4 ChIP‐seq data analysis (GSE226487), suggesting the possibility that SMAD3 and its interacting partner BRD4 form a functional complex on the *RAB4A* TSS. This was corroborated by parallel analyses of histone modifications and ATAC‐seq data, all marking the *RAB4A* TSS with well‐defined peaks of factor binding at this site (Fig. 3D). A similar topology was revealed at the TSSs of the *PDCD6IP* (*ALIX*) and *CD81* genes (Figs S3, S4). In addition, the Hi‐C analysis indicated that the TSSs of the three genes localize in highly interacting regions of the major TADs that map on each respective locus (Fig. 3D and Figs S3, S4). This *in silico* analysis strongly supports the functional data of R‐SMAD silencing and suggests the existence of specific SMAD‐based transcriptional complexes mediating responses of *RAB4A*, *PDCD6IP* (*ALIX*), and *CD81* to TGF‐β signaling.

### TGF‐β enhances ALIX S‐palmitoylation and ALIX‐TSG101 interaction

**Fig. 4:**
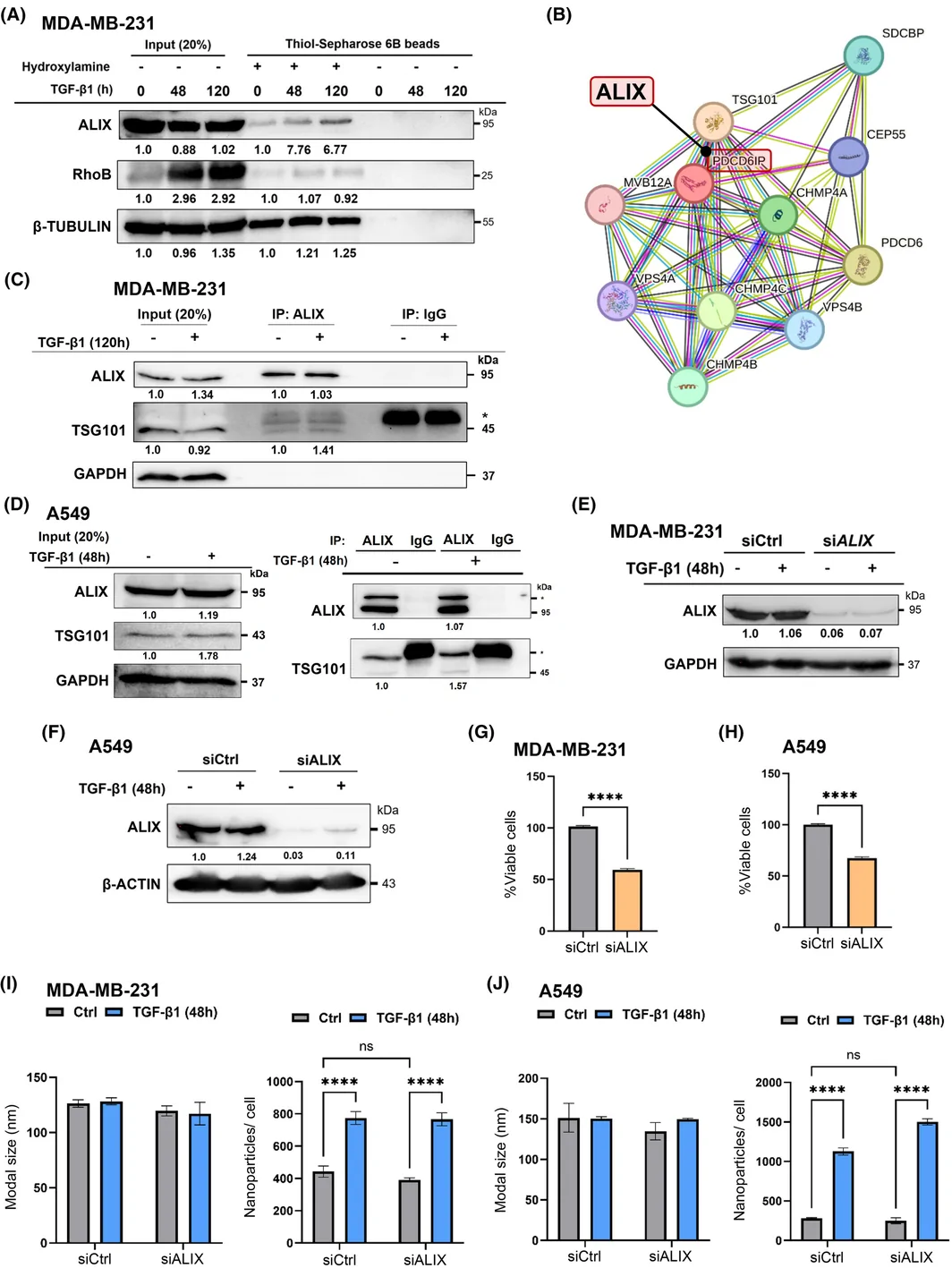
TGF‐β‐induced extracellular vesicle (EV) release is independent of ALIX. (A) Acyl‐resin‐assisted capture assay of ALIX. MDA‐MB‐231 cells were incubated with vehicle (−) or 5 ng·mL^−1^ TGF‐β1 for the indicated time periods and cell lysates were collected (Input). The cell lysates were incubated or not with hydroxylamine prior to pulldown using thiol‐sepharose B beads. Aliquots of the input and the S‐palmitoylation‐enriched proteins were analyzed by SDS/PAGE and immunoblotting for ALIX, RhoB, and β‐TUBULIN, the latter serving as a positive control. (B) Protein–protein interaction network analysis using STRING and a confidence score > 0.7. The protein PDCD6IP (ALIX) and its annotation is clarified in the diagram. (C, D) TGF‐β weakly enhances the protein complex formation between ALIX and TSG101. MDA‐MB‐231 (C) or A549 (D) cells were incubated with vehicle (−) or 5 ng·mL^−1^ TGF‐β1 for 120 h (C) or 48 h (D) and cell lysates were collected (Input). The lysates were immunoprecipitated (IP) with ALIX‐specific antibody or non‐specific IgG, followed by SDS/PAGE and immunoblot with ALIX and TSG101 antibodies. GAPDH immunoblotting serves as a loading control for the input lysates and negative control for the IP. Representative immunoblots of at least two independent biological replicates along with molecular mass markers in kDa are shown. Densitometric values were normalized to the respective control (−) condition. Stars mark the heavy chain of the IgG used for IP. (E, F) Expression levels of ALIX protein with GAPDH or β‐Actin serving as a loading control in MDA‐MB‐231 (E) and A549 (F) cells transiently transfected with control (siCtrl) or specific siRNA targeting ALIX (siALIX). Densitometric values were normalized to the siCtrl. The cells were stimulated with vehicle (Ctrl) or 5 ng·mL^−1^ TGF‐β1 for 48 h. (G, H) Cell viability assay with MDA‐MB‐231 and A549 cells transiently transfected with control (siCtrl) or specific siRNA targeting ALIX (siALIX) for 48 h using PrestoBlue reagent. (I, J) EVs released by MDA‐MB‐231 (I) and A549 (J) cells, which were transiently transfected with siCtrl or siALIX, were quantified by nanoparticle tracking analysis (NTA) in terms of particle modal size (left) and particle number after normalization to the total cell number (right). The cells were stimulated with vehicle (Ctrl) or 5 ng·mL^−1^ TGF‐β1 for 48 h. The cell viability (G, H) and the NTA data (I, J) are presented as mean values of three biological replicates ± SEM, in technical triplicates and p‐values are shown based on unpaired *t*‐test with Welch's correction (G, H) or two‐way ANOVA (I, J), followed by multiple paired comparisons conducted by means of Bonferroni's post‐test method: ****P ≤ 0.0001; ns, not signigicant.

The weak induction of *ALIX* mRNA expression in MDA‐MB‐231 cells did not result in a corresponding upregulation of ALIX protein levels after TGF‐β1 stimulation for 48 or 120 h (Fig. 4A). However, since ALIX function is known to be regulated by post‐translational mechanisms, we examined one such possibility. Of importance, since S‐palmitoylation is a post‐translational modification that affects ALIX interactions with vesicular membranes, offering proximity to other EV regulators during EV biogenesis [22], we sought to explore whether TGF‐β1 stimulation could affect ALIX S‐palmitoylation levels. To this end, we performed an acyl‐resin‐assisted capture (Acyl‐RAC) assay, as previously described [23], while using RhoB and β‐TUBULIN palmitoylation as positive controls for S‐palmitoylated proteins [24]. Our results reveal that TGF‐β1 stimulation for both 48 and 120 h led to a remarkable increase in ALIX S‐palmitoylation in MDA‐MB‐231 cells (Fig. 4A). These data prompted us to investigate whether the effect of TGF‐β signaling on ALIX S‐palmitoylation could impact the protein–protein interaction of ALIX with other regulators of EV biogenesis. Using the Search Tool for the Retrieval of Interacting Genes (STRING) database, members of the ESCRT machinery were among the top proteins interacting with ALIX, including VPS4A, VPS4B, CHMP4A, CHMP4B, CHMP4C, MVB12A, and TSG101 (Fig. 4B). To validate the ALIX‐TSG101 interaction, we conducted co‐immunoprecipitation assays (Figs 4C,D, S5A). Notably, we observed a modest increase in ALIX‐TSG101 complex formation upon TGF‐β1 stimulation for 120 h in MDA‐MB‐231 and for 48 h in A549 cells (Fig. 4C,D). This interaction is consistent with previous reports [25, 26] and supports findings that implicate ALIX‐ESCRT complexes in influencing the sorting of cargo into MVBs [27].

However, whether the enhanced EV release previously observed upon TGF‐β1 stimulation (Fig. 1) could also be driven by S‐palmitoylated ALIX–membrane interactions or ALIX–TSG101 complex formation was an open question. To validate the relevance of ALIX for EV release, we silenced ALIX in MDA‐MB‐231 and A549 cells (Fig. 4E,F). Given that ALIX regulates mitotic spindle orientation, assisting cell division [28], we first confirmed that its depletion significantly reduced cell viability in both cell lines (Fig. 4G,H). Upon stimulation of MDA‐MB‐231 and A549 cells with TGF‐β1 for 48 h, secreted EVs smaller than 200 nm were visualized by TEM, confirming the presence of membrane‐surrounded nanoparticles (Fig. S5B,C), and evaluating their size and number through NTA (Fig. 4I,J) under control and silencing conditions. Importantly, despite the fact that TGF‐β1 stimulation enhanced EV secretion in MDA‐MB‐231 and A549 cells, ALIX silencing did not have an impact on the number of EVs released. These results indicate that ALIX may not be essential for EV release *per se*, but rather might be restricted to the cargo sorting during EV biogenesis, as shown by others [27, 29].

### EV induction by TGF‐β is not CD81‐dependent

**Fig. 5:**
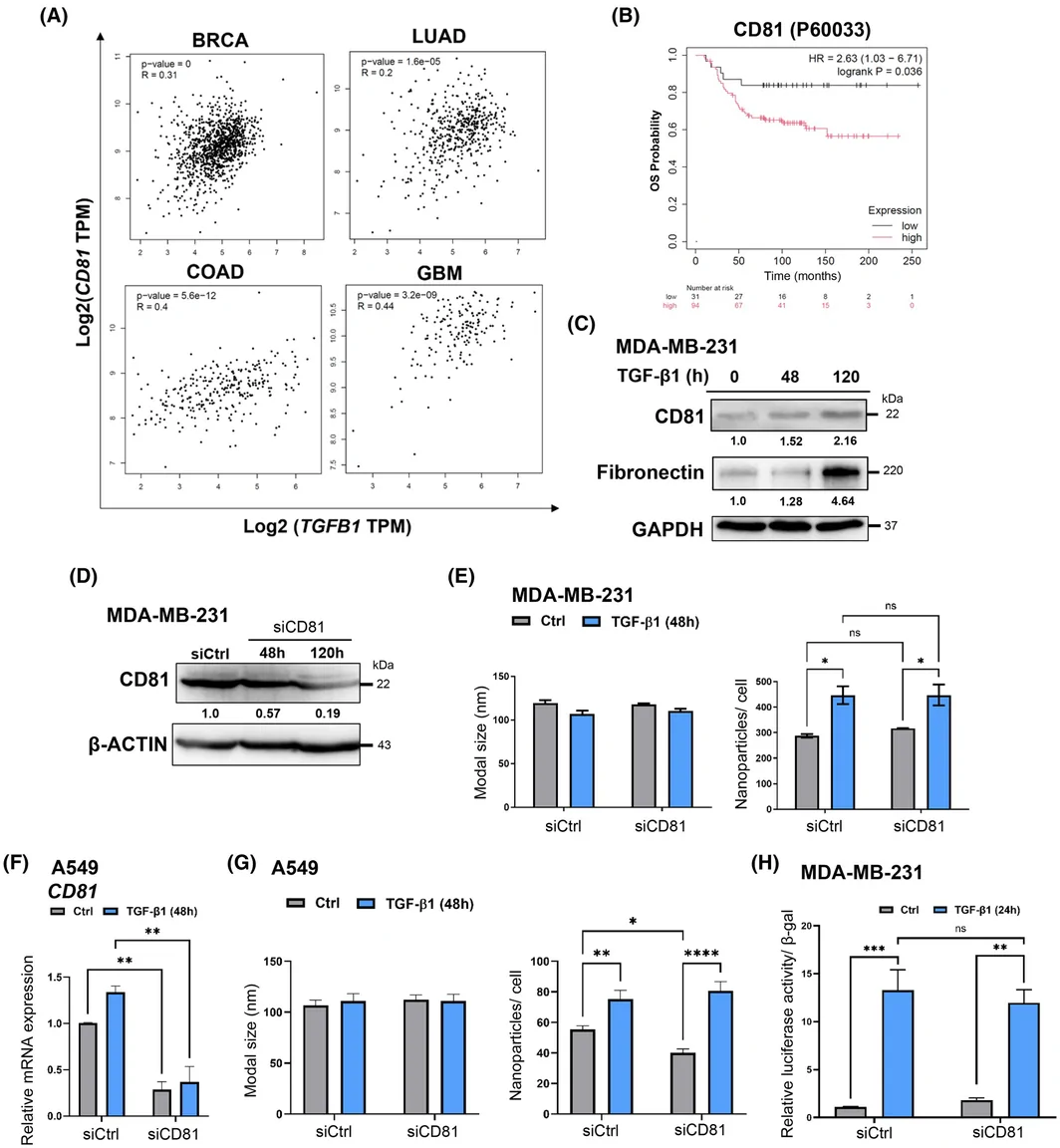
TGF‐β induced extracellular vesicle (EV) release does not rely on CD81. (A, B) Pearson correlation analysis of *CD81* mRNA expression in BRCA, LUAD, COAD, and GBM relative to *TGFB1* mRNA expression, measured as transcripts per million (TPM) transformed by log_2_; data were obtained from The Cancer Genome Atlas (TCGA). *P*‐ and *R*‐values are listed. (B) Higher CD81 protein levels correlate with shorter overall survival (OS) in human BRCA patients. BRCA samples were stratified and compared in the Kaplan–Meier plotter database using auto‐select cutoff. (C) Expression levels of CD81, Fibronectin and GAPDH serving as loading control from MDA‐MB‐231 cells treated with TGF‐β1 for 48 and 120 h. Representative immunoblots of at least two independent biological replicates, along with molecular mass markers in kDa, are shown. Densitometric values were normalized to the vehicle control. (D) Expression levels of CD81 protein with β‐Actin serving as loading control in MDA‐MB‐231 cells transiently transfected with control (siCtrl) or specific siRNA targeting CD81 (siCD81). Note that 81% of CD81 silencing was observed only after 120 h of transfection. Densitometric values were normalized to the siCtrl. (E, G) EVs released by MDA‐MB‐231 (E) and A549 (G) cells transiently transfected with control or siRNA targeting *CD81*, quantified by nanoparticle tracking analysis (NTA) in terms of modal particle size (left) and particle number after normalization to the total cell number (right). The cells were stimulated with vehicle (Ctrl) or 5 ng·mL^−1^ TGF‐β1 for 48 h. (F) RT‐qPCR analysis of *CD81* mRNA in A549 cells after transient transfection with siCtrl or si*CD81* after stimulation of the transfected cells with 5 ng·mL^−1^ TGF‐β1 for 48 h. Values represent fold‐change of mRNA expression normalized to GAPDH and expressed relative to the level at siCtrl. (H) Relative luciferase activity generated in MDA‐MB‐231 cells transiently transfected with siCtrl or siCD81 by additional transfection of the TGF‐β‐inducible CAGA_12_‐luc reporter, normalized to β‐galactosidase activity generated by a co‐transfected reporter, after stimulation of the cells or not with 5 ng·mL^−1^ TGF‐β1 for 24 h. The data in E–H are presented as mean values of three biological replicates ± SEM, in technical triplicates and *P*‐values are shown based on two‐way ANOVA, followed by multiple paired comparisons conducted by means of Bonferroni's post‐test method: *P ≤ 0.05; **P ≤ 0.01; ***P ≤ 0.001; ****P ≤ 0.0001; ns, not significant.

In line with TGF‐β inducing *ALIX* and *CD81* mRNA expression, we found a positive and significant correlation between *TGFB1* mRNA and the expression of these genes in different tumor types across TCGA datasets (Figs 5A, S5D). Additionally, ALIX and CD81 protein expression were further correlated with clinical features using BRCA patient datasets. Only high CD81 expression significantly correlated with the poor overall survival of BRCA patients (Fig. 5B), while no statistical difference was noted among ALIX differential expression (Fig. S5E). Furthermore, we validated the previous mRNA expression analysis by confirming enhanced CD81 protein levels in MDA‐MB‐231 cells upon TGF‐β1 stimulation for 48 and 120 h (Fig. 5C), using Fibronectin as a positive TGF‐β‐responsive control.

The induction of CD81 protein expression by TGF‐β1 prompted us to determine the effect of silencing the *CD81* gene on TGF‐β‐induced EV secretion. CD81 knockdown in MDA‐MB‐231 cells (Fig. 5D, note the reduction of CD81 expression only after 120 h of transfection) did not affect the amount of EVs secreted by MDA‐MB‐231 cells in comparison to the respective controls in the absence or presence of TGF‐β1 stimulation (Fig. 5E), which is in line with previous findings in lymphoblasts and HeLa cells [30, 31]. However, CD81 silencing in A549 cells (Fig. 5F) significantly reduced EV secretion (Fig. 5G). In the presence of TGF‐β1, despite reduced CD81 levels, a significant increase in secreted A549 EVs was observed (Fig. 5G). The integrity of the EVs enriched in the VSF was assessed by TEM and found to be largely intact (Fig. S5F). These results suggest that CD81 might primarily play a role in cargo sorting during EV biogenesis under TGF‐β signaling activation. This is consistent with previous reports demonstrating that tetraspanins contribute to the selective recruitment of proteins into EVs [32] and aligns with our recent findings that the major factor regulating EV secretion upon TGF‐β activation is MAPK‐dependent, followed by cholesterol biosynthesis, rather than SMAD‐dependent [7]. Given that tetraspanins CD9 and CD81 have been reported to facilitate the association of TGFβR1 with TGFβR2 in melanoma cells [33], we evaluated TGF‐β signaling using the CAGA_12_‐luciferase reporter, which measures TGF‐β receptor‐induced SMAD transcriptional activity, in MDA‐MB‐231 cells following CD81 silencing (Fig. 5H). Unexpectedly, TGF‐β–induced reporter activation was not significantly affected by CD81 knockdown compared with controls, suggesting that CD81 alone may not affect TGF‐β receptor activation.

### RAB4A‐mediated short‐loop feedback restricts TGF‐β‐induced EV secretion

**Fig. 6:**
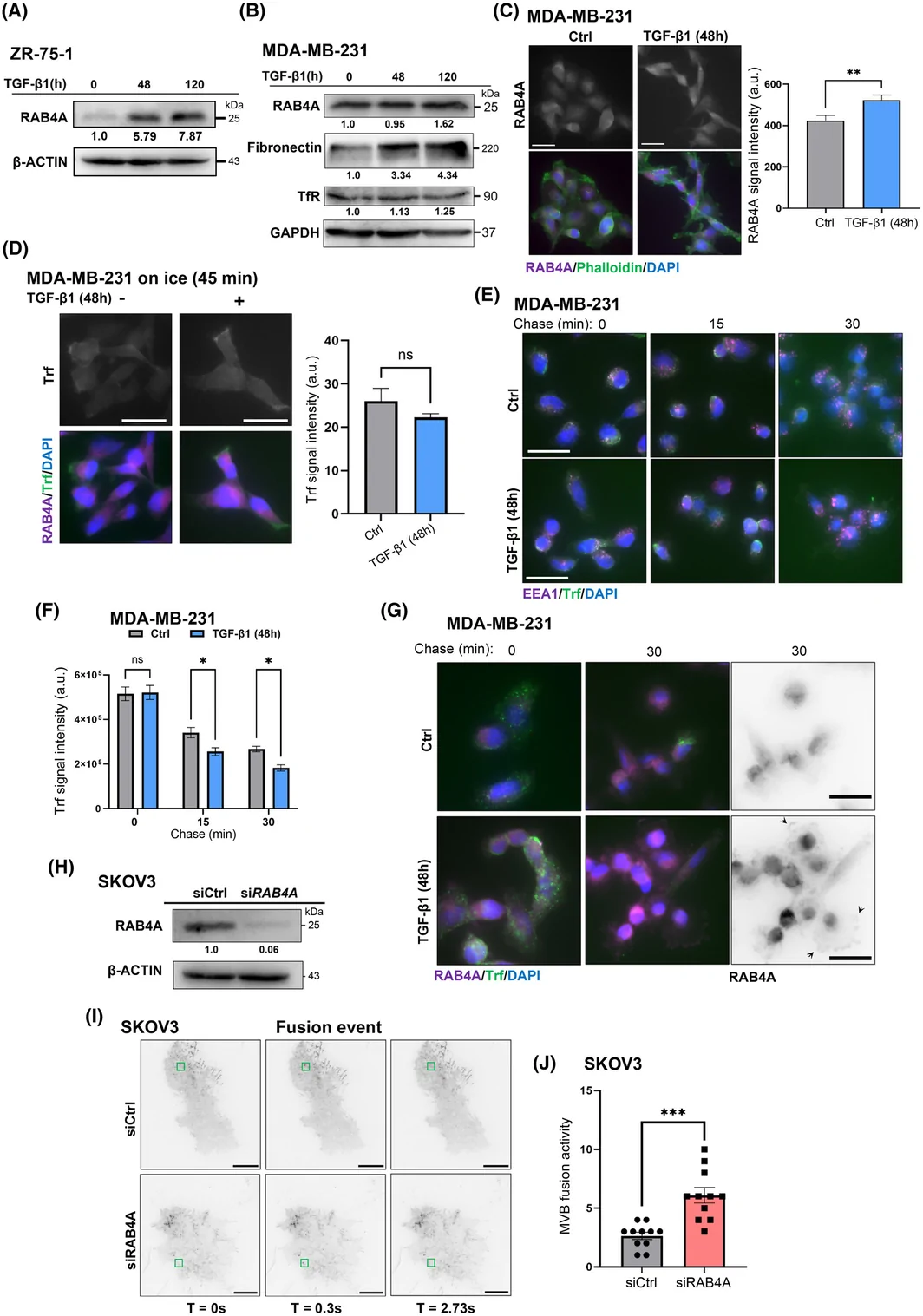
RAB4A‐mediated recycling limits multivesicular body (MVB)–plasma membrane fusion. (A, B) Expression levels of RAB4A and β‐Actin or GAPDH serving as loading control from ZR‐75‐1 (A) and MDA‐MB‐231 (B) cells treated with TGF‐β1 for 48 and 120 h. Representative immunoblots of at least two independent biological replicates, along with molecular mass markers, in kDa are shown. Densitometric values were normalized to the vehicle control. (C) Representative immunofluorescence microscopy pictures of MDA‐MB‐231 cells stimulated or not with 5 ng·mL^−1^ TGF‐β1 for 48 h. RAB4A (magenta), phalloidin (green), and nuclei (DAPI; blue) are labeled; scale bar = 50 μm. (D) Representative immunofluorescence microscopy pictures of MDA‐MB‐231 cells stimulated or not with 5 ng·mL^−1^ TGF‐β1 for 48 h and incubated on ice for 45 min with Alexa488‐conjugated Transferrin (Trf, green). RAB4A (magenta) and nuclei (DAPI; blue) are labeled; scale bar = 50 μm. (E, F) Representative immunofluorescence microscopy pictures of MDA‐MB‐231 cells stimulated or not with 5 ng·mL^−1^ TGF‐β1 for 48 h. Alexa488‐conjugated Transferrin (Trf, green) chased with unlabeled holo‐Trf for the indicated times. The early endosome antigen protein 1 (EEA1, magenta) and nuclei (DAPI; blue) are labeled; scale bar = 50 μm. (G) Representative immunofluorescence microscopy pictures of MDA‐MB‐231 cells stimulated or not with 5 ng·mL^−1^ TGF‐β1 for 48 h. Alexa488‐conjugated Trf (green) chased with unlabeled holo‐Trf for the indicated times. RAB4A (magenta) and nuclei (DAPI; blue) are labeled; scale bar = 50 μm. Cell fluorescence intensity in C–E was measured using imagej with *n* of cells ≥ 10 per condition from at least two biological replicates. (H) Expression levels of RAB4A protein with β‐Actin serving as loading control in SKOV3 cells transfected transiently with control (siCtrl) or specific siRNA targeting *RAB4A* (si*RAB4A*). Densitometric values were normalized to the siCtrl. (I) Representative TIRF microscopy images showing CD63‐pHluorin fusion events over a 3‐min time (*T*) course in SKOV3 cells transiently transfected with siCtrl or siRAB4A. Green squares indicate fusion events; scale bar = 50 μm. (J) Quantification of MVB fusion events over a 3‐min time course in SKOV3 cells transiently transfected with control (siCtrl) or specific siRNA targeting *RAB4A* (si*RAB4A*). Data represent *n* ≥ 10 cells per condition from two independent biological replicates. Data in panels C, D, F, and J are presented as mean ± SEM from at least two independent biological replicates. *P*‐values in panels C, D, and J were calculated using an unpaired *t*‐test with Welch's correction. *P*‐values in panel *F* were determined by two‐way ANOVA, followed by multiple paired comparisons conducted by means of Bonferroni's post‐test method: *P ≤ 0.05; **P ≤ 0.01; ***P ≤ 0.001; ns, not significant.

RAB4A regulates endosomal trafficking and recycling of proteins, and it has been suggested to limit EV secretion by increasing the recycling of early endosomes and consequently reducing the maturation of MVBs [7, 34, 35, 36]. RAB4A protein analysis by immunoblot and immunofluorescence microscopy indicated a higher accumulation of this protein in ZR‐75‐1 and MDA‐MB‐231 cells upon TGF‐β1 stimulation for 48 and even higher at 120 h (Fig. 6A–C), validating the previous mRNA expression analysis (Fig. 1F). Thus, we wondered whether endosomal recycling, in general, could be regulated by TGF‐β signaling, and to this aim, we tested whether Transferrin (Trf) receptor recycling, a well‐established protein studied in endocytosis and recycling studies that is also regulated by a RAB4 ‘short‐loop’ [35], could be affected by TGF‐β signaling. To this end, MDA‐MB‐231 cells stimulated or not with TGF‐β1 for 48 h were incubated with Alexa488‐conjugated Trf on ice for 45 min (Fig. 6D). Unbound labeled Trf was washed away, and the cells were shifted to 37 °C for 15 min to allow internalization. Subsequently, cells were returned to ice for 45 min and treated with a quenching agent to eliminate any surface‐bound Alexa488 fluorescence signal that could have remained. This was followed by incubation with complete media supplemented with holo‐Trf and with the visualization of chased Trf receptor retained in early endosomes by fluorescence imaging of the early endosome antigen 1 (EEA1) and considered as the starting point in recycling (0 min, Fig. 6E,F). After a 30‐min chase at 37 °C, most of the internalized Trf receptors were recycled back to the plasma membrane, as evidenced by the shedding of labeled Trf signals (Fig. 6E,F). This recycling was particularly pronounced in cells pretreated with TGF‐β1 for 48 h, suggesting that TGF‐β‐induced RAB4A might affect Trf receptor recycling dynamics. To further validate this hypothesis, we repeated the Trf internalization assay in MDA‐MB‐231 cells, this time assessing colocalization with RAB4A (Fig. 6G). Interestingly, cells pretreated with TGF‐β1 for 48 h exhibited a remarkable increase in RAB4A‐positive compartments at the cell periphery after 30 min, suggesting an acceleration of endosomal recycling. These results suggest that TGF‐β1 stimulation promotes RAB4A expression and subsequently faster trafficking of Trf receptor through RAB4A‐associated recycling compartments. Moreover, to elucidate the mechanism by which RAB4A limits EV secretion, we employed a tetraspanin CD63‐based pH‐sensitive reporter (CD63‐pHluorin) to monitor MVB–plasma membrane fusion events using live total internal reflection fluorescence (TIRF) microscopy [37]. The results demonstrated that depletion of RAB4A in SKOV3 cells (Fig. 6H) significantly enhances the frequency of MVB–plasma membrane fusion, indicating that RAB4A‐mediated fast endosomal recycling reduces MVB maturation and fusion with the plasma membrane (Fig. 6I,J).

**Fig. 7:**
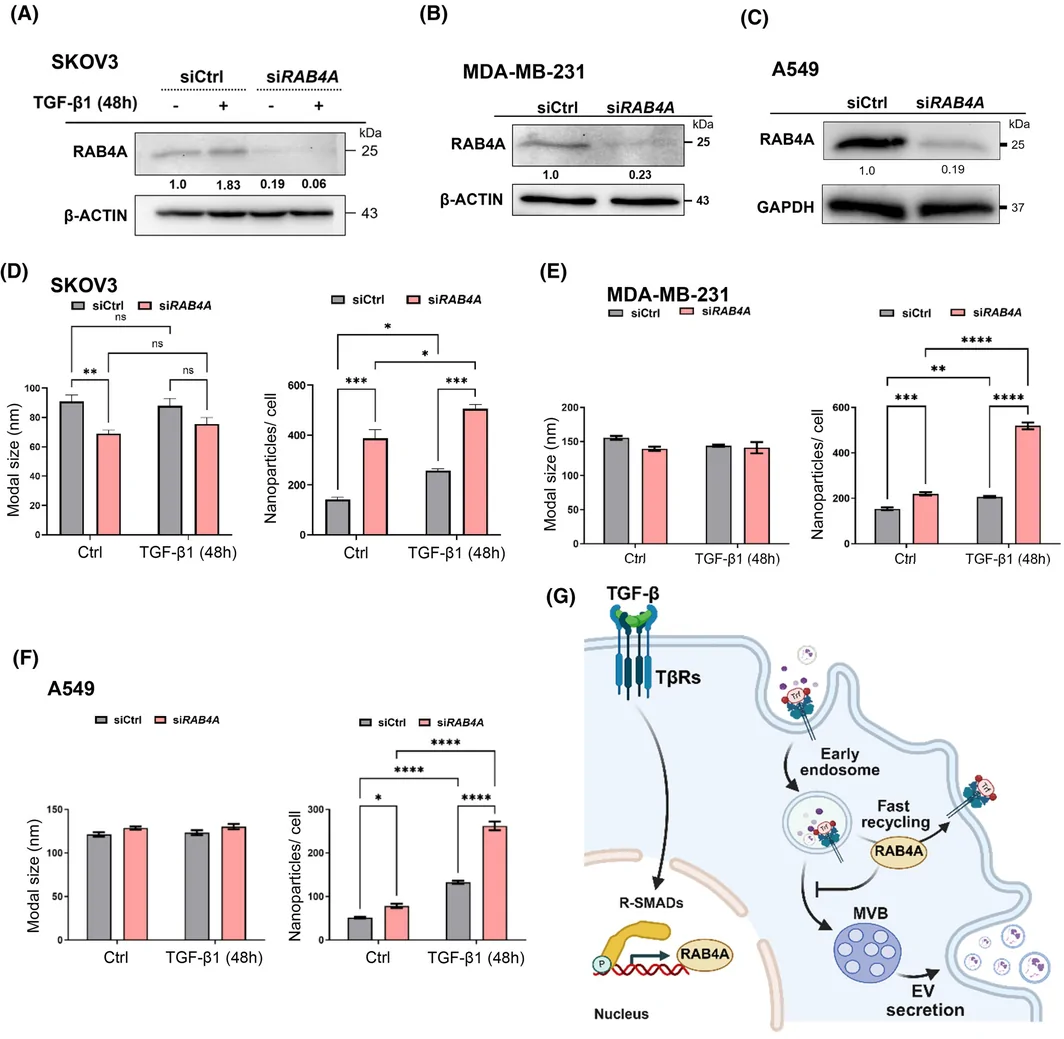
TGF‐β‐induced RAB4A acts as a negative regulator of extracellular vesicle (EV) release. (A–C) Expression levels of RAB4A protein with β‐Actin or GAPDH serving as loading control in SKOV3 (A), MDA‐MB‐231 (B), and A549 (C) cells transfected transiently with control (siCtrl) or specific siRNA targeting *RAB4A* (si*RAB4A*). Densitometric values were normalized to the siCtrl. (D–F) EVs released by SKOV3 (D), MDA‐MB‐231 (E), and A549 (F) cells transfected transiently with control or siRNA targeting *RAB4A*, quantified by nanoparticle tracking analysis (NTA) in terms of modal particle size (left) and particle number after normalization to the total cell number (right). The cells were stimulated with vehicle (Ctrl) or 5 ng·mL^−1^ TGF‐β1 for 48 h. The NTA data (D–F) are presented as mean values of three biological replicates ± SEM, in technical triplicates and p‐values are shown based on two‐way ANOVA, followed by multiple paired comparisons conducted by means of Bonferroni's post‐test method: *P ≤ 0.05; **P ≤ 0.01; ***P ≤ 0.001; ****P ≤ 0.0001; ns, not significant. (G) Schematic model summarizing the effect of RAB4A‐mediated recycling limiting TGF‐β‐induced EV release. TGF‐β from the extracellular matrix binds to its receptors (TβRs) on the plasma membrane, activating receptor‐regulated SMADs (R‐SMADs). In the nucleus, R‐SMAD enhances expression of *RAB4A*. This small GTPase increases fast recycling of early endosomes, exemplified by the Trf receptor, limiting EV secretion as shown in this paper. Created in Biorender.com. Moustakas, A. (2026) https://BioRender.com/co1vrpt.

Finally, to investigate the potential role of RAB4A in TGF‐β‐induced EV release, we silenced RAB4A in SKOV3 (note the increased levels of RAB4A upon TGF‐β1 stimulation), MDA‐MB‐231, and A549 cells, and subsequently isolated EVs (Fig. 7A–F), whose morphology was characterized by TEM (Fig. S5G–I). Silencing RAB4A significantly enhanced EV secretion in all three cell models compared with siCtrl‐transfected cells, supporting the enhanced MVB–plasma membrane fusion and consequent exosomal release. Moreover, after RAB4A silencing, TGF‐β1 stimulation further amplified the number of secreted EV from both cells (Fig. 7D–F), suggesting that RAB4A physiologically limits the mechanism of EV biogenesis induced by TGF‐β. In other words, RAB4A, which enhances early endosomal recycling in response to TGF‐β1, might function as a negative feedback mechanism that restrains TGF‐β‐induced EV secretion via the MEK/cholesterol pathway.

## Discussion

These findings make a major new contribution to the field of EV biology by demonstrating that the small GTPase RAB4A can play a negative role in controlling EV secretion in response to TGF‐β signaling (Fig. 7G). This report follows a small number of very recent papers that, for the first time, address the cell biological processes through which the multifaceted cytokine TGF‐β regulates EV biogenesis. Accordingly, we reported that TGF‐β activates a MEK1 kinase‐dependent pathway that activates SREBP2, which upregulates several key enzymes in the cholesterol biosynthetic pathway; out of these enzymes the rate‐limiting *dehydrocholesterol reductase* (*DHCR7*) was found to be transcriptionally regulated and functionally important for inducing EV release in cancer cell models by TGF‐β [7]. This mechanism is compatible with other reports that have previously established the enhanced secretion of EVs carrying TGF‐β [38] and induced by biological contexts, such as EMT and invasive, pro‐metastatic cancer cell behavior [1], all known to be potently induced by TGF‐β signaling [4, 39]. In addition, fibroblast EV release has been established to be positively regulated by TGF‐β signaling, as is the case of cancer‐associated fibroblasts [8] and non‐senescent fibroblasts [9]. Yet, based on the context‐dependent actions of TGF‐β signaling, it may not be surprising that human pulmonary artery smooth muscle cells that produce EVs under hypertensive conditions did not exhibit specific responses to TGF‐β signaling that affected EV secretion [10], and even negative effects of TGF‐β on EV secretion have been reported in breast cancer cell cultures, the latter involving a SMAD‐mediated signaling input that suppresses expression of the RAB27B small GTPase [11]. These limited cases indicate that the mechanisms driven by TGF‐β may be diverse and complex and motivated this study which aimed to identify additional regulatory steps in this interesting aspect of cancer cell biology.

TGF‐β/EV cell biology mirrors the general function of this signaling pathway since several cell responses to TGF‐β involve a combination or distinct modules that involve SMAD signaling, signaling by ubiquitin ligases, MEK/ERK, PI3K/AKT, and other protein or lipid kinases [6, 40]. To this end, we searched systematically for various signaling mediators based on the association of expression of secretory and EV biogenetic genes in various human tumors, queried in the data of cancer patient repositories. This approach led us to examine specific key players of the three major pathways that have been established as contributors to EV biogenesis, beyond lipid and cholesterol biosynthesis [2]. Using a combination of inhibitory approaches, MEK1 or AKT kinase inhibition and SMAD‐specific silencing during TGF‐β signaling, we screened for responses of ESCRT, RAB, and tetraspanin family members and retrieved more meaningful regulation for three specific genes: *PDCD6IP*/*ALIX* that encodes for an ESCRT adaptor protein, the tetraspanin *CD81*, and the small GTPase *RAB4A* gene. Based on our recent report that defined MEK1 kinase/SREBP2 activity as being important for DHCR7/cholesterol‐mediated EV secretion [7], we selected these genes as they exhibited a distinct response to TGF‐β that was insensitive to MEK1 kinase inhibition but sensitive to SMAD2 and SMAD3 silencing.

In addition to the gene expression modulation, we also examined post‐translational aspects of regulation and for this matter focused on the adaptor protein ALIX [41]. ALIX and its interaction with TSG101 have been established as an important biochemical step in EV biogenesis that is guided by the ESCRT machinery [22, 42, 43]. Accordingly, we analyzed the S‐palmitoylation of ALIX and its physical interaction with TSG101 and found that TGF‐β signaling could affect both ALIX S‐palmitoylation and formation of the ALIX‐TSG101 complex. However, since functional assays after silencing ALIX did not perturb the potency of TGF‐β signaling in inducing EV secretion, we were prohibited from capitalizing further on an ALIX‐centric mechanism. It is worth discussing that the transcriptional induction of ALIX by TGF‐β was not only dependent on SMAD2/3 but also on AKT kinase signaling inputs. This potentially warrants further studies on the transcriptional signals that regulate ALIX gene expression, a postulate that is supported even by the analysis of Hi‐C, ChIP‐seq and ATAC‐seq data on the *PDCD6IP‐DT*/*ALIX* gene. The latter indicated not only SMAD3 binding to the *PDCD6IP‐DT*/*ALIX* gene chromatin, but also of the chromatin remodeler BRD4 and associated histone modifications, which partially depend on signaling inputs, such as AKT. Thus, analysis of ESCRT‐mediated functions deserves deeper investigation in the context of TGF‐β signaling.

The tetraspanin CD81 [44] exhibited reproducible regulation at the mRNA and protein level by TGF‐β signaling, primarily in the TNBC model of MDA‐MB‐231 cells. Similar to ALIX, *CD81* mRNA induction by TGF‐β required both SMAD2/3 and AKT signaling inputs, a topic for future analysis, also supported by the chromatin‐based analysis of histone modifications and SMAD3 binding. Interestingly, TGF‐β‐induced EV release was unaffected by CD81 silencing in both MDA‐MB‐231 and A549, although basal EV secretion was reduced in A549 cells. These data indicate a strong dependency of CD81 regulation on cell type and biological context [30, 31]. Since CD81, like the other tetraspanins (eg., CD9 and CD63) regulate curvature of bilayered lipid vesicles and their function in EV biogenesis is linked to the cholesterol content of the bilayer, it is possible that CD81, which also supports the association among TGF‐β receptors [33], can be upregulated by TGF‐β in some cancer cells in order to serve as a cofactor to the TGF‐β/cholesterol pathway [7]. However, silencing of CD81 alone did not impact TGF‐β signaling activation in MDA‐MB‐231 cells. In other words, the functional role of CD81 downstream of TGF‐β may involve cooperation with other tetraspanins and with the generation of cholesterol and its incorporation into vesicles during EVs biogenesis.

Our final focus addressed the regulation and function of RAB4A, an enzyme with well‐established roles during vesicular trafficking, driving the fast recycling of cargo proteins from endosomes to the plasma membrane [45, 46, 47]. This established role of RAB4A that associates with early endosomes and is implicated in recycling of diverse proteins was also corroborated in our system, as TGF‐β enhanced the endocytic recycling of the Trf receptor. Unlike *ALIX* and *CD81*, the induction of *RAB4A* mRNA expression by TGF‐β was dependent on SMAD2/3 but not on AKT signaling. This does not preclude that additional kinase inputs may pair with the SMAD signals in regulating *RAB4A* expression, a postulate that is supported by the Hi‐C, ChIP‐seq and ATAC‐seq analysis of SMAD3 association and histone modifications on the *RAB4A* locus. A related transcriptional mechanism has been ascribed to *RAB5A*, which is upregulated by TGF‐β/SMAD4 signaling in MDA‐MB‐231 cells. SMAD4 then induces the TFEB, which subsequently binds to the TCACGTG motif in the *RAB5A* promoter, thereby activating its expression [48]. Additionally, *RAB4A* expression in T cells has been shown to be regulated by oxidative stress through redox‐sensitive TFs, such as NRF1, USF1, PSIP1, and IRF2 [49, 50]. Given that TGF‐β promotes ROS generation via NOX4 in glioblastoma cells [51], this could suggest that *RAB4A* expression could be influenced by the interplay between TGF‐β signaling and oxidative stress–responsive pathways. Notwithstanding, the focus on RAB4A generated the most unexpected result of this study, whereby RAB4A silencing enhanced the fusion of MVBs with the plasma membrane, followed by EV secretion under basal and TGF‐β‐inducible conditions. This effect was linked to the reduced recycling of endocytosed vesicular cargo back to the plasma membrane, suggesting that the RAB4A‐based mechanism must be at the core of EV biogenesis that stems from MVBs. It must be emphasized that the potency of secreted EVs was particularly strong under TGF‐β stimulation conditions, since cancer cells release significantly higher numbers of EVs upon RAB4A silencing. Based on these data, we propose that the transcriptional induction of *RAB4A* by TGF‐β signaling forms a negative feedback loop, which aims at counteracting the positive effect of TGF‐β‐mediated cholesterol accumulation and EV secretion.

The details of this model deserve deeper investigation. The phenotype uncovered here resembles an equivalent effect measured in *Drosophila* motor neurons, whereby silencing of retromer, the endosomal complex that directs retrograde vesicular transport to the Golgi apparatus, led to EV accumulation [52]. However, genetic ablation of *Rab4* or *Rab11* in such neurons in the fly led to a reduction in EV cargo proteins, suggesting a more complex mechanism of vesicular regulation in which the Rab4/Rab11 recycling pathways crosstalk with the retromer machinery, resulting in balanced loading of cargo into EVs. On the contrary, our finding is in agreement with several established functions of RAB4A that preferentially direct vesicular trafficking and cargo content to the plasma membrane via the so‐called recycling route by organizing multiprotein complexes and their association with the microtubular system [36, 46, 47]. Thus, it is logical to suggest that TGF‐β, by regulating RAB4A expression, may aim at enhancing recycling of proteins, and consequently, the pool of endocytic vesicles that is driven toward EV secretion becomes limited. Notably, a potential mechanism involving RAB4A, ALIX, and CD81 in TGF‐β‐regulated EV secretion can relate to the selective loading of EV cargo [7]. The evidence presented here stimulates follow‐up studies on the multiprotein machinery that drives the balance between endosomal recycling to the plasma membrane and the generation of exosomes from MVBs, processes that likely have relevance beyond cancer biology. Aberrant endosomal dynamics and dysregulated EV biogenesis are increasingly implicated in systemic autoimmune diseases, such as systemic lupus erythematosus (SLE), where altered EV release contributes to heightened inflammatory signaling [53]. Given that TGF‐β is essential for regulatory T‐cell (Treg) differentiation, and that this pathway is compromised in SLE and related autoimmune diseases [54], our findings suggest that alterations in RAB4A‐dependent TGF‐β–EV responses could impact mechanisms of immune tolerance. Future studies elucidating this mechanism in both cancer and immune cell populations will probably illuminate broader physiological and pathological roles of this trafficking circuit in shaping TGF‐β‐dependent regulation of small EV secretion [7, 10, 11].

In conclusion, this study paves the way for more in‐depth investigations of specific components of vesicular trafficking and EV biogenesis, whose regulation is affected by TGF‐β signaling. A more holistic approach that will target the complete vesicular proteome and lipidome is warranted in order to understand the complex dynamics that orchestrate EV secretion under the signaling inputs of cytokines like TGF‐β.

## Materials and methods

### Cell culture and treatments

The cell lines MDA‐MB‐231 (HTB‐26: human basal‐TNBC), ZR‐75‐1 (CRL‐1500: luminal BRCA), MCF7 (HTB‐22: luminal BRCA), and A549 (CCL‐185: LUAD) were obtained from the American type culture collection (https://www.atcc.org/). The ovarian serous cystadenocarcinoma cells SKOV3 and OVCAR3 were a generous gift from Anzhelika Vorobyeva, Uppsala University, Sweden. The cell lines were cultured in Dulbecco's modified Eagle's medium (DMEM; Thermo Fisher Scientific, Stockholm, Sweden) or Roswell Park Memorial Institute‐1640 (RPMI; Thermo Fisher Scientific) and supplemented with 10% fetal bovine serum (FBS; Thermo Fisher Scientific), 100 U·mL^−1^ penicillin, and 100 μg·mL^−1^ streptomycin (Merck, Stockholm, Sweden). The non‐tumorigenic breast epithelial MCF10A cells were cultured in DMEM/F12 (Thermo Fisher Scientific), supplemented with 5% horse serum (Biowest, Almeco A/S, Esbjerg, Denmark), 20 ng·mL^−1^ epidermal growth factor (Thermo Fisher Scientific), 100 ng·mL^−1^ cholera toxin, 0.5 μg·mL^−1^ hydrocortisone, 10 μg·mL^−1^ insulin (Merck). The cells were kept in a humidified incubator at 37 °C and 5% CO_2_ and constantly checked for mycoplasma presence and authenticated through STR profiling (Eurofins, Uppsala, Sweden). The cells were kept under starvation for at least 12 h in FBS‐free media prior to treatment for different time periods with 5.0 ng·mL^−1^ recombinant human TGF‐β1 (Thermo Fisher Scientific). When indicated, the cells were treated with inhibitors U0126 (MEKi, no.: 19‐147; Merck) or Capivasertib (AKTi, no.: TA9H11E417D2; Merck). Drug or siRNA‐mediated toxicity was assessed using the PrestoBlue HS Cell Viability Reagent (no.: P50200; Thermo Fisher Scientific) that was applied to the cells for 48 h, as previously described [7]. As vehicle, 0.1% dimethyl‐sulfoxide (DMSO) was used and the data are presented as units of fluorescence emitted from the treated cells after normalization with the fluorescence emitted by the DMSO‐treated cells. Dose–response curves were generated for the chemical inhibitors. Experiments were performed in triplicates and data are expressed as mean ± SEM.

### EV isolation and characterization

EV isolation and characterization was performed as previously described [7]. The cells were incubated for 48 h in DMEM or RPMI supplemented with 10 μg·mL^−1^ insulin (Sigma‐Aldrich AB, Stockholm, Sweden), 1× MEM non‐essential amino acids (no.: 11140‐050), 10 μg·mL^−1^ fibroblast growth factor‐basic (no.: 13256‐029) and 1 mm sodium pyruvate (no.: 11360‐039) (all from Thermo Fisher Scientific), in the presence or absence of TGF‐β1. The conditioned medium was centrifuged at 1200× ***g*** for 5 min to clear cell debris, following additional centrifugation at 4000× ***g*** for 5 min, while measuring the corresponding number of cells. The VSF was filtered through a 0.2 μm filter and concentrated 20× by tangential flow filtration on a 100 kDa Ultra‐15 Centrifugal Filter (Amicon; Merck) by centrifugation at 4000× ***g***.

The small EVs enriched in the VSF were assessed using NTA (NanoSight system; Malvern Panalytical, Malvern, UK) to characterize the nanoparticle concentration and size distribution. Modal diameter and the ratio of nanoparticles per cell were analyzed statistically. Furthermore, the presence of EVs in the VSF was performed through TEM, by mixing the VSF with an equal volume of 4% w/v formaldehyde and incubating with uranyl oxalate, pH 7.0, for 5 min, then stained in a drop containing 4% w/v uranyl acetate and 2% w/v methylcellulose, as described [55]. Images were acquired using a Tecnai™ G2 Spirit BioTwin TEM (Thermo Fisher Scientific/FEI, Stockholm, Sweden) operated at 80 kV or an HT7800 TEM (Hitachi, Ibaraki, Japan) operated at 100 kV. Images obtained with the Tecnai™ system were captured using an ORIUS SC200 CCD camera, while those acquired with the HT7800 were captured using a Xarosa EMSIS camera and subsequently processed with the gatan digital micrograph software (Gatan Inc./Blue Scientific, Pleasanton, CA, USA). Finally, 150 μL of VSF were incubated with 40 μL of anti‐CD81 magnetic Dynabeads™ (no.: 10616D; Thermo Fisher Scientific) for 60 min at 37 °C to fractionate EV subpopulations based on CD81 presence. The CD81‐positive EVs bound to the magnetic beads were immobilized, washed with PBS, lysed with RIPA buffer (50 mm Tris–HCl pH 7.5, 150 mm NaCl, 1% NP‐40, 0.5% sodium deoxycholate and 0.1% SDS) and resolved by SDS/PAGE.

### Correlation analysis of gene expression

Using RNA‐seq data from cancer patients available through GEPIA2, we performed correlation analysis of *TGFB1* mRNA co‐expression with GSEA hallmark signatures for protein secretion and with the gene ontologies of GOBP for EV biogenesis (Table S1) across multiple cancer types, including BRCA, LUAD, OV, HNSCC, COAD, GBM, LIHC, and PAAD. Co‐expression was assessed based on the Pearson correlation statistic (*R* > 0.2, *P*< 0.05). Overall survival in breast cancer patients was predicted using the KM plotter protein database (KMplot) [56].

### RNA extraction and RT‐qPCR

RNA extraction and real‐time RT‐qPCR were performed as previously described [57]. Relative gene expression levels were quantified after normalization to *GAPDH* levels. Primer sequences of the analyzed genes are listed in Table S5.

### Immunoblotting and immunoprecipitation

Immunoblotting was performed as previously described [57]. Briefly, the lysis buffer was supplemented with protease inhibitors (no.: 11697498001; Merck). The proteins were analyzed by SDS/PAGE either directly or following immunoprecipitation using 2.5 μg of the respective primary antibody (described in Table S6), captured with Dynabeads™ M‐280 Sheep Anti‐Mouse IgG (no.: 11202D; Thermo Fisher Scientific). imagej bundled with Java 1.8.0_172 (National Institutes of Health, Bethesda, MD, USA) was used to normalize intensity levels according to the expression of loading control proteins GAPDH, β‐ACTIN, or β‐TUBULIN.

### MOTIF analysis

The motif enrichment analysis was performed using Hypergeometric Optimization of Motif EnRichment (HOMER; http://homer.ucsd.edu/homer/motif/) suite v4.11. The *de novo* motif search was performed in a genomic region spanning ±1 kb from the gene's transcription start site and the enriched motifs were sorted based on the false discovery rate. Additionally, TF profiling was also done using the Enrichr database, which integrates experimentally validated TF binding data from JASPAR (https://jaspar.elixir.no/) and ChIP Enrichment Analysis (ChEA3: https://maayanlab.cloud/chea3/) databases to identify enriched TF.

### Genome data analysis

Hi‐C datasets from A549 (GSE92819) cells were processed using hicexplorer (version 3.7.2) as previously described [58]. Paired‐end reads were aligned to the GRCh38/hg38 reference genome using bowtie2. Contact matrices were generated using hicbuildmatrix for A549, followed by removal of invalid read pairs. Matrices were normalized with hicNormalize using the ‘smallest’ method and corrected with hicCorrectMatrix (ICE). TADs were identified with hicFindTads at 3‐kb resolution. ChIP‐seq datasets (GSE92782, GSE51510, GSE226487, and CRA001325) were aligned to GRCh38/hg38 using bowtie2, and peaks were called using macs2 under default parameters. Hi‐C contact maps and ChIP‐seq tracks were visualized with pygenometracks (version 3.9).

### Acyl‐resin‐assisted capture

The Acyl‐RAC assay that removes acyl groups from cysteine residues with hydroxylamine, followed by direct conjugation of the free cysteines by a thiol‐reactive resin, was performed with minor modifications as described [59]. Briefly, 1 × 10^7^ cells per sample were lysed in 500 μL of 50 mm Tris, pH 7.5, 150 mm NaCl, 5 mm EDTA, 10% v/v glycerol, 1% v/v Triton X‐100, supplemented with complete protease inhibitors (no.: 11697498001; Roche Diagnostics Scandinavia AB, Bromma, Sweden) and 50 nm of N‐ethylmaleimide (no.: 04259; Fluka/Honeywell Fisher Scientific, Gothenburg, Sweden), under rotation for 2 h at 4 °C. An aliquot of 50 μL was stored as input, while 200 μL were incubated with 0.75 m hydroxylamine (HA; no.: 159417; Merck) or with PBS for 2 h at room temperature, followed by incubation with 30 μL of epoxy‐activated thiol‐sepharose 6B (Merck). After overnight incubation, the beads were washed with lysis buffer, after which, proteins were desorbed by 50 μL of PBS and resolved by SDS/PAGE. In the presence of hydroxylamine, ALIX was detected in the thiol‐reactive resin pulldown fraction, but not in the negative control that was not treated with hydroxylamine.

### Protein–protein interaction network analysis

A protein–protein interaction network for proteins interacting with ALIX was constructed using the Search Tool for the Retrieval of Interacting Genes database (string‐version 11.5; http://string‐db.org/), with the required high confidence score (> 0.7).

### siRNA transfections

Transfections were performed using siLentFect (no.: 170‐3360; Bio‐Rad Laboratories Inc., Sundbyberg, Sweden) and the respective siRNA (20 nm), according to the manufacturer. The cells were collected for knockdown validation 48 h after transfection and seeded for additional experiments. We used distinct human‐specific siRNA oligonucleotides against each target mRNA (Table S7).

### Immuno‐ and direct fluorescence microscopy

The cells were seeded in an 8‐well slide followed by treatments with TGF‐β1 for the indicated time periods. Immunofluorescence was performed as previously described [7]. Fixed cells were incubated with primary antibodies in 1% BSA dissolved in PBS overnight at 4 °C, followed by incubation with Alexa‐Fluor‐488 or Alexa‐Fluor‐546 secondary antibodies at a dilution of 1 : 500 in PBS for 1 h at room temperature. The antibodies are listed in Table S6. Cell nuclei were stained with 4′,6‐Diamidino‐2‐phenylindole dihydrochloride (DAPI; Merck) at a dilution of 1 : 1000 in PBS for 10 min at room temperature. The cells were examined using a Nikon Eclipse 90i microscope (Nikon Europe B.V., Amstelveen, the Netherlands) equipped with a CCD camera (Andor multi pixel sCMOS camera, Oxford Instruments, Abingdon, UK). At least 10 random pictures were taken with 60× objectives at the same exposure time.

### Luciferase assays

Cells were transiently transfected with Lipofectamine 3000 (Thermo Fisher Scientific) using the CAGA_12_‐luciferase promoter‐reporter and treated with TGF‐β1 for 24 h. Luciferase activity was measured using the firefly and Renilla dual‐luciferase assay kit (Biotium, Fremont, CA, USA), as previously described [7]. Normalized luciferase activity is reported as the mean ± SEM from triplicate measurements.

### Transferrin receptor recycling assay

The cells were incubated under starvation media overnight prior to the assay. On ice, the cells were treated with 5 μg·mL^−1^ of Alexa Fluor™ 488‐conjugated Trf (T13342; Thermo Fisher Scientific) for 45 min, washed twice with PBS, and returned to 37 °C in order to allow Trf internalization. After 15 min, the cells were returned to ice and treated for 45 min with media containing 10% FBS, 25 μg·mL^−1^ unlabeled holo‐Trf (T4132; Merck) and anti‐Alexa fluor® 488 (no.: A11094; Thermo Fisher Scientific) in order to quench the remaining surface signal. Then, the cells were washed twice with PBS and incubated in complete media at 37 °C. At the indicated times, the cells were fixed with 4% w/v paraformaldehyde in PBS for 15 min at room temperature.

### CD63‐pHluorin and TIRF microscopy

The cells were seeded on 25‐mm glass coverslips (Menzel‐Gläser, no.: 1) in six‐well plates and allowed to adhere overnight. The next day, cells were transfected with the respective siRNA as described above. Forty‐eight hours later, the cells were transfected with Lipofectamine 3000 (Thermo Fisher Scientific) using 500 ng of pCMV‐Sport6‐CD63‐pHluorin [37]. After 24 h, the coverslips were placed in an open Sykes‐Moore perfusion chamber as exchangeable bottoms and mounted on the stage of a custom‐built TIRF microscopy setup based around an inverted Eclipse Ti‐E microscope (Nikon) equipped with a TIRF illuminator and a 60×/1.45 NA Plan‐Apo objective (Nikon). CD63‐pHluorin was excited at 491 nm using a diode‐pumped solid‐state laser (Cobolt; Hübner Photonics, Solna, Sweden) and emission light was separated by a 527/27 interference filter (Semrock, Rochester, NY, USA) mounted in a motorized filter wheel (Lambda 10‐3; Sutter Instruments, Novato, CA, USA), with a shutter (Sutter Instruments) blocking the beam between image acquisitions. Fluorescence was captured by an Orca‐ER CCD camera (Hamamatsu) at 3.33 Hz and controlled by the MetaFluor software (Molecular Devices Corp., Downington, PA, USA). All experiments were conducted at 37 °C in complete culture medium. Time‐lapse sequences were acquired at 300 ms intervals for a total duration of 3 min, resulting in 601 frames, with one moderately expressing CD63‐pHluorin–positive cell in the field of view per acquisition. Images were analyzed offline using the fiji software. Briefly, the fusion events were manually identified by visually inspecting time‐lapse TIRF sequences frame‐by‐frame. Events were classified as MVB‐PM fusion when they exhibited a fluorescence burst followed by exponential decay, had a circular shape, displayed minimal movement after the burst, and persisted for at least three frames from peak intensity until fluorescence returned to background levels. MVB fusion activity was quantified as the number of events per cell per time‐lapse recording.

### Statistical analyses

All data (except when otherwise indicated) are presented as the mean ± SEM. Comparisons were performed using one‐way or two‐way ANOVA, followed by multiple paired comparisons conducted by means of the Tukey's or Bonferroni's post‐test method when applicable. Additional statistical methods are explained in individual methods and figure legends. The data were analyzed with graphpad prism 10.4 (GraphPad Software, San Diego, CA). A *P*‐value < 0.05 was necessary to determine all statistically significant differences.

## Conflict of interest

The authors declare no conflict of interest.

## Author contributions

**DMR‐J** contributed to the conceptualization, resources, experimental design, funding acquisition, methodology, data acquisition, validation, visualization, data analysis, data interpretation, writing—original draft, writing—revision, and review and editing. **MAD** contributed to the methodology, data acquisition, visualization, and writing—review and editing. **HF** and **OI‐H** contributed to the methodology, data acquisition, and visualization. **JB** contributed to the methodology and visualization. **AM** contributed to the resources, data interpretation, funding acquisition, supervision, project administration, writing—original draft, writing—revision, and review and editing.

## Supporting information


**Table S1.** GSEA pathways signature and GOBP database.
**Table S2.** Motif analysis of transcription factors on the *CD81* promoter sequence.
**Table S3.** Motif analysis of transcription factors on the *PCDP6IP* (*ALIX*) promoter sequence.
**Table S4.** Motif analysis of transcription factors on the *RAB4A* promoter sequence.
**Table S5.** Primer sequences used for RT‐qPCR assays.
**Table S6.** List of antibodies used in this study.
**Table S7.** List of siRNAs used in this study.
**Fig. S1.** TGF‐β regulates the expression of genes involved in EV biogenesis.
**Fig. S2.** TGF‐β‐induced *CD81*, *ALIX*, and *RAB4A* mRNA expression requires R‐SMAD signaling.
**Fig. S3.** Genomic landscape of the *ALIX* locus in A549 cells.
**Fig. S4.** Genomic landscape of the *CD81* locus in A549 cells.
**Fig. S5.** Related to Figs 4, 5, and 7. TGF‐β triggers EV release via an ALIX and CD81‐independent pathway.

## Data Availability

The data that support the findings of this study are available from the corresponding author (dorival.mrj@imbim.uu.se) upon reasonable request.
