# Supplementary material for: RAB4A acts as a negative feedback regulator of extracellular vesicle secretion during TGF‐β signaling
Source: FEBS J. 2026 Mar 30;293(17):5340–62. doi: 10.1111/febs.70520 (PMC13534978; doi:10.1111/febs.70520)
Supplement: Supplementary file 1 [file FEBS-293-5340-s001.pdf]

RAB4A acts as a negative feedback regulator of extracellular vesicle secretion during TGF-β signaling

Dorival Mendes Rodrigues-Junior[1#], Maria Anastasia Doumani[1], Hao Fu[2], Jelle van den Bor[3], Olof Idevall-Hagren[4], Aristidis Moustakas[1]

SUPPLEMENTARY FILES

Supplementary Tables

Supplementary Table S1. Gene Set Enrichment Analysis (GSEA) pathways signature and gene ontology aspects of biological process (GOBP) database.

**GSEA: protein secretion**							
ABCA1	ADAM10	ANP32E	AP1G1	AP2B1	AP2M1	AP2S1	AP3B1
AP3S1	ARCN1	ARF1	ARFGAP3	ARFGEF1	ARFGEF2	ARFIP1	ATP1A1
ATP6V1B1	ATP6V1H	ATP7A	BET1	BNIP3	CAV2	CD63	CLCN3
CLN5	CLTA	CLTC	COG2	COPB1	COPB2	COPE	CTSC
DNM1L	DOP1A	DST	EGFR	ERGIC3	GALC	GBF1	GLA
GNAS	CXCL1	CXCL12	CXCL6	CXCL8	DAB2	DCN	DKK1
GOLGA4	GOSR2	ICA1	IGF2R	KIF1B	KRT18	LAMP2	LMAN1
M6PR	MAPK1	MON2	NAPA	NAPG	OCRL	PAM	PPT1
RAB14	RAB22A	RAB2A	RAB5A	RAB9A	RER1	RPS6KA3	SCAMP1
SCAMP3	SCRN1	SEC22B	SEC24D	SEC31A	SGMS1	SH3GL2	SNAP23
SNX2	SOD1	SSPN	STAM	STX12	STX16	STX7	TMED10
TMED2	TMX1	TOM1L1	TPD52	TSG101	TSPAN8	USO1	VAMP3
VAMP4	VAMP7	VPS45	VPS4B	YIPF6	YKT6	ZW10	

**GOBP: extracellular vesicle biogenesis**							
ARRDC1	ARRDC4	ATP13A2	ATP9A	CD34	CHMP2A	CHMP3	CHMP6
COPS5	HGS	IFNG	PDCD6IP	PRKN	RAB7A	RAB11A	RAB27A
SDC1	SDC4	SDCBP	SMPD3	SNF8	STAM	TSG101	VPS4A
VPS4B							

Supplementary Table S2. Motif analysis of significant predicted transcription factors with DNA binding motifs on the *CD81* promoter sequence.

**ChEA3**							
AF4	AR	ATF3	BCL6	CBP	CHD1	CREB1	CTCF
E2A	ELK3	ENL	FLI1	HAND2	KDM2B	KLF4	LXR
MAF	MITF	MYB	NFKB1	P63	RUNX1	SMAD3	TCF21
TFAP2C	UBF1/2	VDR	XRN2				
**JASPAR**							
GATA2	MAX	NR3C1	NKX2-8	MYC	USF2	USF1	MZF1 1-4
**HOMER**							
ARNT:AHR	BREU	EOMES	FOSB::JUN	GRHL1	HBP1	HOXB4	MSANTD3
MSX2	NFIC	NKX3-1	RXRA	SNAI2	TBX5	ZBTB12	ZBTB18
ZFP187	ZFP691	ZIC2	ZKSCAN5				

Supplementary Table S3. Motif analysis of significant predicted transcription factors with DNA binding motifs on the *PCDP6IP (ALIX)* promoter sequence.

**ChEA3**							
AF4	AR	BRD4	CDX2	CHD1	CLOCK	CSB	CTBP2
CTCF	EBNA1	ENL	ESR1	FLI1	FOXA1	FOXA2	FOXM1
FOXP1	GABP	GATA1	GATA2	ILF3	JUN	KLF5	MITF
MYC	MYCN	NCOR1	NFKB1	NRF2	P300	PAX3-FKHR	PKCTHETA
PPARD	RUNX1	SMAD2/3	SMAD3	SMAD4	SOX2	TCF3/E2A	TCF4
TFEB	TP63	UBF1/2	VDR	WDR5	WT1	YY1	
**JASPAR**							
ETS1	FOXC1	FOXJ1	HOXA5	MEF2A	NFE2	NKX3-1	NRF1
TBP	YY1	ZBTB16					
**HOMER**							
DMRT1	ETV4	HIF-1a	Homez	HOXA9	HOXC9	IRF5	NFIC
NPAS4	NR1H4	OSR1	PBX1	PKNOX2	PRDM14	PTF1A	RARA
RHOX11	RXRA	SNAI2	SOX12	SOX17	SOX30	YY2	ZBED1
ZFP410	ZDP57	ZIC3	ZKSCAN1	ZNF341	ZNF7		

Supplementary Table S4. Motif analysis of significant predicted transcription factors with DNA binding motifs on the *RAB4A* promoter sequence.

**ChEA3**							
AHR	ARNT	ASH2L	CEBPB	CREB1	E2F4	EP300	ERG
ESRRB	FOXA2	FOXO3	GATA1	GATA2	GATA4	HNF4A	KDM5B
LMO2	LYL1	MEF2A	MEIS1	MITF	MYCN	ANOG	NUCKS1
POU5F1	PRDM14	REST	SALL4	SMAD2	SMAD3	SMARCA4	SOX2
SOX9	SPI1	STAT3	TAL1	TCF4	TCF7	TET1	TFCP2L1
TP53	TP63	ZFX					
**JASPAR**							
CEBPB	ETV4	FOS	FOXC1	GATA2	JUND	LEF1	NR5A2
RBPJ	SNAI1	SNAI2	TCF3	USF2			
**HOMER**							
BARHL2	CDX4	CHOP	DMRT1	E2F2	EBF2	EHF	HNF6B
LEF1	MAFF	MEF2A	MYB	NEUROG2	NFAT	NR1H4	OCT6
POU2F3	POU5F1	RBPJ	RFX3	RFX4	RORA	SMAD4	SOX1
SRF	STAT6	TCF7	TEAD3	TFAP4	VENTX	YY2	ZBTB3
ZFP187	ZFP691	ZNF317	ZNF449	ZSCAN29			

Supplementary Table S5. Primer sequences used for RT-qPCR assays.

Gene Symbol	Forward (F) and Reverse (R) Primers Sequences (5'-3')
ARF6	F - ATGGGGAAGGTGCTATCCAAAATC<br>R - GCAGTCCACTACGAAGATGAGACC
CD9	F - GCATTGCCGTGGTCATGA<br>R - TGCGGATAGCACAGCACAAG
CD63	F - CCCGAAAAACAACCACACTGC<br>R - GATGAGGAGGCTGAGGAGACC
CD81	F - TGATCCTGTTTGCCTGTGAG<br>R - CAGTTGAGCGTCTCATGGAA
FN1 **(Fibronectin)**	F - CCCAGACTTATGGTGGCAATTC<br>R - AATTTCCGCCTCGAGTCTGA
GAPDH	F - GGAGTCAACGGATTTGGTCGTA<br>R - GGCAACAATATCCACTTTACCA
PDCD6IP **(*ALIX*)**	F - GACGCTCCTGAGATATTATGATCAGA<br>R - ACACACAGCTCTTTTCATATCCTAAGC
RAB4A	F - GACCTGGATGCAGATCGTGA<br>R - TCAAGGCAGCATCTCCGTAC
RAB5A	F - ACGGGCCAAATACGGGAAAT<br>R - TCAAACTTTACCCCAATGGTACTC
RAB27A	F - GCTTTGGGAGACTCTGGTGTA<br>R - TCAATGCCCACTGTTGTGATAA
SERPINE1 **(PAI1)**	F - GAGACAGGCAGCTCGGATTC<br>R - GGCCTCCCAAAGTGCATTAC
SMAD2	F - TGGCTGGCACCCTGCAACAG<br>R - TGCCTTCGGTATTCTGCTCCCCA
SMAD3	F - GCAATATTCCAGAGACCCCACC<br>R - TAGGTTTGGAGAACCTGCGTCC
TSG101	F - TCCAGTCTTCTCTCGTCCTATTTC<br>R - TTTCCTCCTTCATCCGCCATCTC

Supplementary Table S6. List of antibodies used with dilution factors and application.

Antibody	Application	Manufacturer and Cat. #
**Anti-ALIX**	Immunoblotting (1:250) Immunoprecipitation 2.0 µg	SantaCruz Biotechnology #sc-53540
**Anti-CD81**	Immunoblotting (1:250)	SantaCruz Biotechnology #sc-166029
**Anti-EEA1**	Immunofluorescence (1:200)	BD Biosciences #610456
**Anti-Fibronectin (FN1)**	Immunoblotting (1:1,000) Immunofluorescence (1:150)	Millipore #F3648
**Anti-GAPDH**	Immunoblotting (1:1,000)	ThermoFisher Scientific #AM4300
**Anti-GM130**	Immunoblotting (1:1,000)	ThermoFisher Scientific # MA5-48468
**Anti-PAI1**	Immunoblotting (1:1,000)	BD Biosciences #612025
**Anti-RAB4**	Immunoblotting (1:1,000) Immunofluorescence (1:150)	BD Biosciences #610888
**Anti-RhoB**	Immunoblotting (1:1,000)	SantaCruz Biotechnology #sc-180
**Anti-SMAD2**	Immunoblotting (1:1,000)	Cell Signaling #5339S
**Anti-SMAD2/3**	Immunoblotting (1:1,000)	BD Biosciences #610843
**Anti-Transferrin receptor/CD71**	Immunoblotting (1:1,000)	Cell Signaling #13113
**Anti-TSG101**	Immunoblotting (1:250)	SantaCruz Biotechnology #sc-136111
**Anti-β-ACTIN**	Immunoblotting (1:1,000)	SantaCruz Biotechnology #sc-69879
**Anti-β-TUBULIN**	Immunoblotting (1:1,000)	SantaCruz Biotechnology #sc-101527

Supplementary Table S7. List of siRNAs used in this study.

siRNA	Product ID
**SMARTpool: ON-TARGETplus SMAD2**	Dharmacon; L-003561-00-0005
**SMARTpool: ON-TARGETplus SMAD3**	Dharmacon; L-020067-00-0005
**ON-TARGETplus non-targeting pool**	Dharmacon; D-001810-10-20
**FlexiTube GeneSolution – ALIX**	Qiagen; 1027416: SI03119354
**FlexiTube GeneSolution - DHCR7**	Qiagen; 1027416: SI00363300
**Negative Control siRNA**	Qiagen; 1022076
**Silencer® Select – RAB4A**	ThermoFisher Scientific; s11767
**Silencer® Select Negative Control**	ThermoFisher Scientific; 4390843

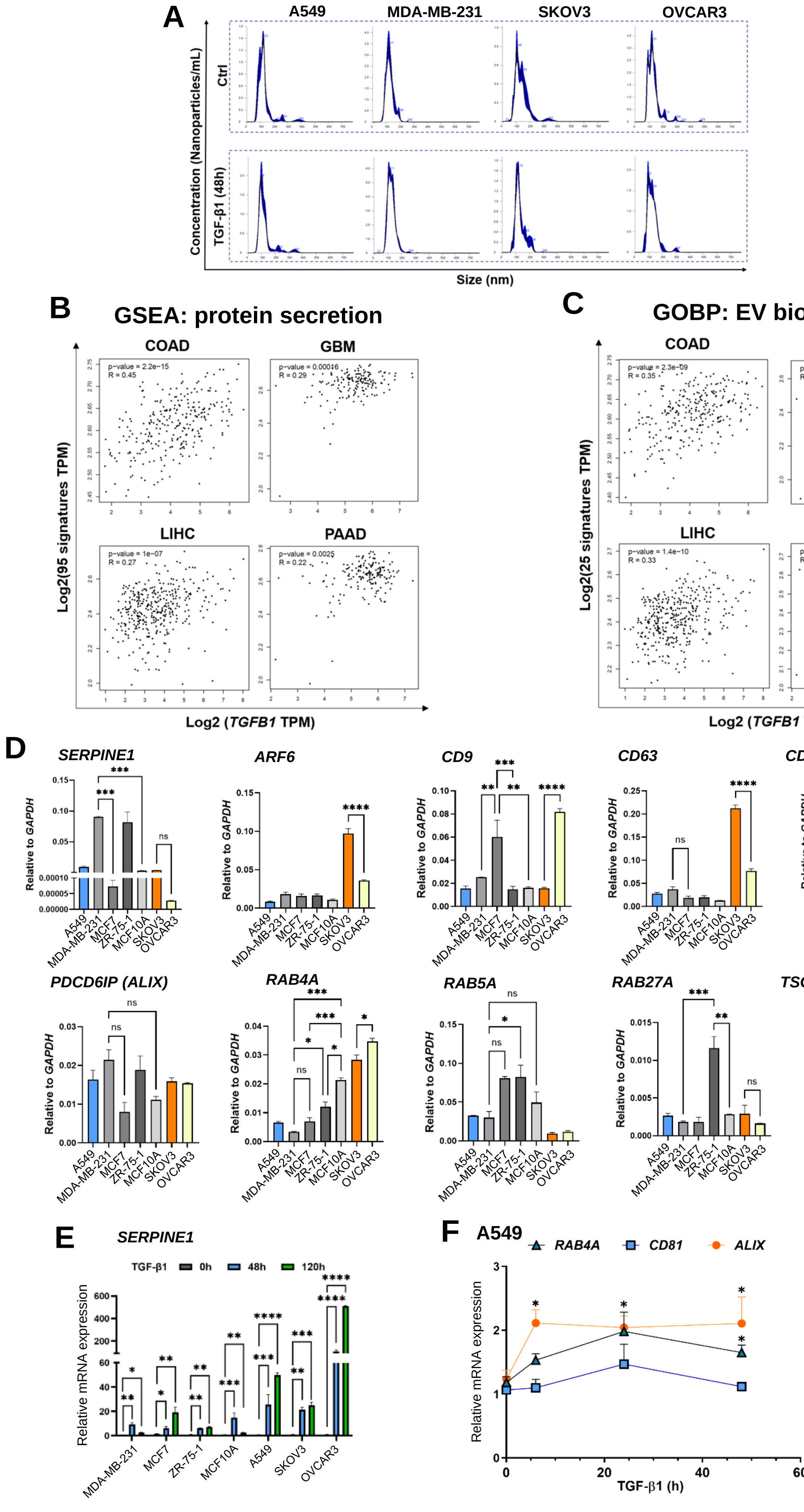
A
A549
MDA-MB-231
SKOV3
OVCAR3
Ctrl
TGF-β1 (48h)
Concentration (Nanoparticles/mL)
Size (nm)
B
GSEA: protein secretion
COAD
GBM
LIHC
PAAD
Log2(95 signatures TPM)
Log2 (TGFB1 TPM)
C
GOBP: EV biogenesis
COAD
GBM
LIHC
PAAD
Log2(25 signatures TPM)
Log2 (TGFB1 TPM)
D
SERPINE1
ARF6
CD9
CD63
CD81
PDCD6IP (ALIX)
RAB4A
RAB5A
RAB27A
TSG101
Relative to GAPDH
E
SERPINE1
TGF-β1 0h 48h 120h
Relative mRNA expression
F
A549
RAB4A
CD81
ALIX
Relative mRNA expression
TGF-β1 (h)

Suppl. Fig S1

Supplementary Figure S1. Related to Figure 1. TGF-β regulates the expression of genes involved in EV biogenesis. (A) Representative nanoparticle traces measured by NTA. The VSF from A549, MDA-MB-231, SKOV3 and OVCAR3 cells stimulated or not with 5 ng/mL TGF-β1 for 48 hours were analyzed by NTA 3.4 software, according to the manufacturer's protocol. 100nm beads were used as a positive control. **(B, C)** Pearson correlation analysis of mRNA expression in COAD, GBM, LIHC and PAAD of gene signatures for protein secretion (B) and extracellular vesicles biogenesis (C) relative to the *TGFB1* mRNA expression, measured as transcripts per million (TPM) transformed by $\log_2$; data were obtained from TCGA. P- and R-values are listed. **(D)** RT-qPCR analysis of the indicated genes in A549, MDA-MB-231, MCF7, ZR-75-1, MCF10A, SKOV3 and OVCAR3 cells. Values represent fold-change of mRNA expression normalized to *GAPDH*. **(E)** RT-qPCR analysis of *SERPINE1* mRNA levels in MDA-MB-231, MCF7, ZR-75-1, MCF10A, A549, SKOV3 and OVCAR3 cells upon stimulation with 5 ng/mL TGF-β1 for 48 or 120 hours. Values represent fold-change of mRNA expression normalized to *GAPDH* and expressed relative to the level at 0 h TGF-β1 (Ctrl). **(F)** RT-qPCR analysis of the indicated mRNA levels (*RAB4A*, *CD81* and *ALIX*) in A549 cells upon stimulation with 5 ng/mL TGF-β1 for 6, 24 and 48 hours. Values represent fold-change of mRNA expression normalized to *GAPDH* and expressed relative to the unstimulated control level (Ctrl). Data in D-F are presented as mean values of three biological replicates ± SEM, each in technical triplicates. P-values in panel D and F were determined by one-way ANOVA followed by multiple paired comparisons conducted by Bonferroni's post-test method while in panel E is shown based on two-way ANOVA, followed by multiple paired comparisons conducted by means of Bonferroni's post-test method. p-values: $^*p \leq 0.05$; $^{**}p \leq 0.01$; $^{***}p \leq 0.001$; $^{****}p \leq 0.0001$.

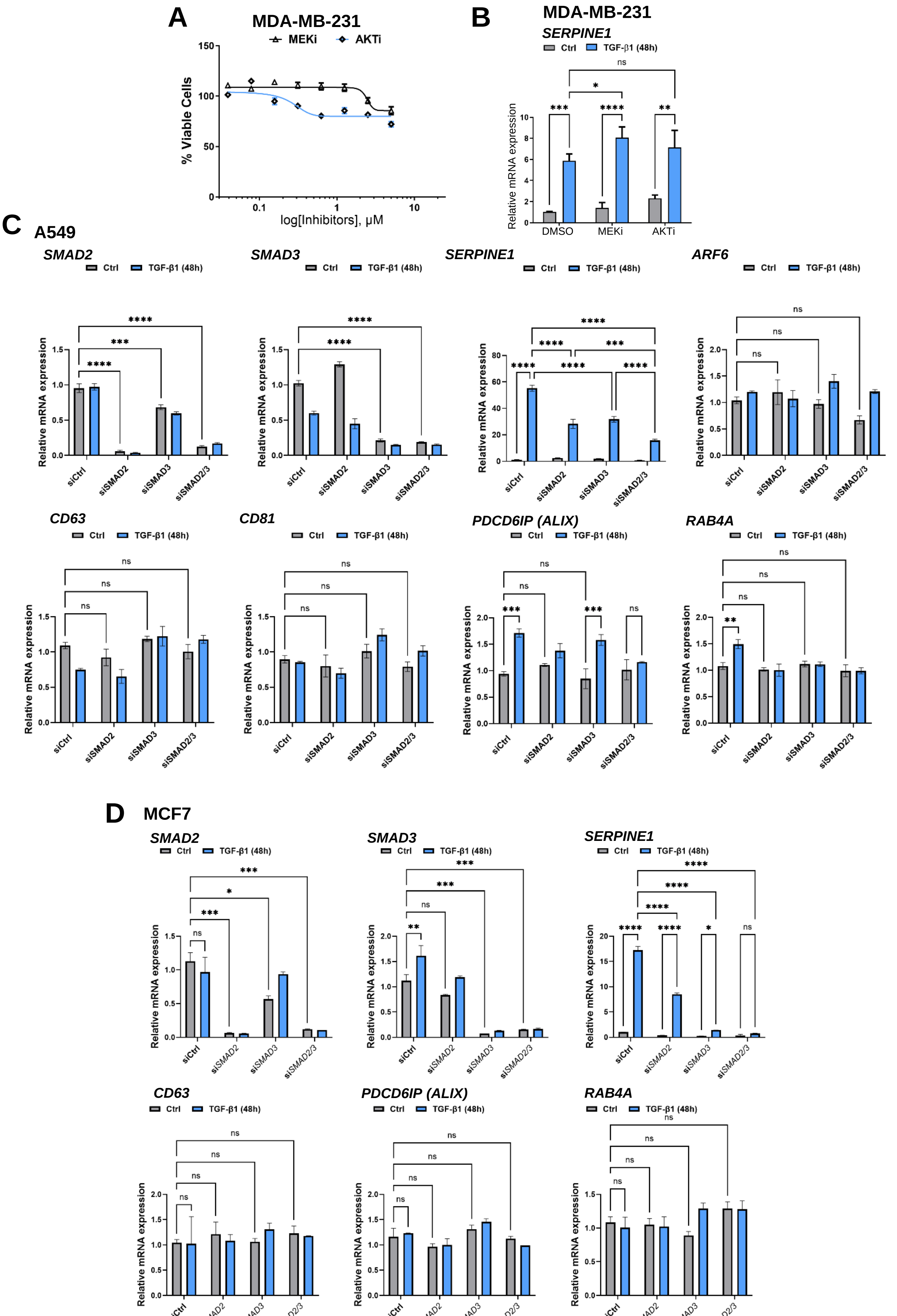
A
MDA-MB-231
MEKi
AKTi
% Viable Cells
log[Inhibitors], μM
B
MDA-MB-231
SERPINE1
Ctrl
TGF-β1 (48h)
Relative mRNA expression
DMSO
MEKi
AKTi
C
A549
SMAD2
SMAD3
SERPINE1
ARF6
CD63
CD81
PDCD6IP (ALIX)
RAB4A
siCtrl
siSMAD2
siSMAD3
siSMAD2/3
D
MCF7
SMAD2
SMAD3
SERPINE1
CD63
PDCD6IP (ALIX)
RAB4A

Suppl. Fig S2

Supplementary Figure S2. Related to Figure 2. TGF-β-induced *CD81*, *ALIX* and *RAB4A* mRNA expression requires R-SMAD signaling. (A) Cell viability curves of MDA-MB-231 cells in the presence of increasing concentrations of MEKi or AKTi (logarithmic scale). The data are presented as mean values of three biological replicates ± SEM, each in technical triplicates. **(B)** RT-qPCR analysis of *SERPINE1* mRNA levels in MDA-MB-231 cells stimulated with vehicle (Ctrl) or 5 ng/mL TGF-β1 for 48 hours in the absence (DMSO vehicle, Ctrl) or presence of 5 µM MEKi or 1 µM AKTi. **(C, D)** RT-qPCR analysis of the indicated mRNAs in A549 (C) and MCF7 (D) cells after transient transfection with siCtrl, SMAD2-specific, SMAD3-specific siRNAs or their combination after stimulation of the transfected cells with 5 ng/mL TGF-β1 for 48 hours. Values represent fold-change of mRNA expression normalized to *GAPDH* and expressed relative to the level at siCtrl. The data in B-D are presented as mean values of at least two biological replicates ± SEM, in technical triplicates and p-values are shown based on two-way ANOVA, followed by multiple paired comparisons conducted by means of Tukey's post-test method: p-values: *p ≤ 0.05; **p ≤ 0.01; ***p ≤ 0.001; ****p ≤ 0.0001; ns, not significant.

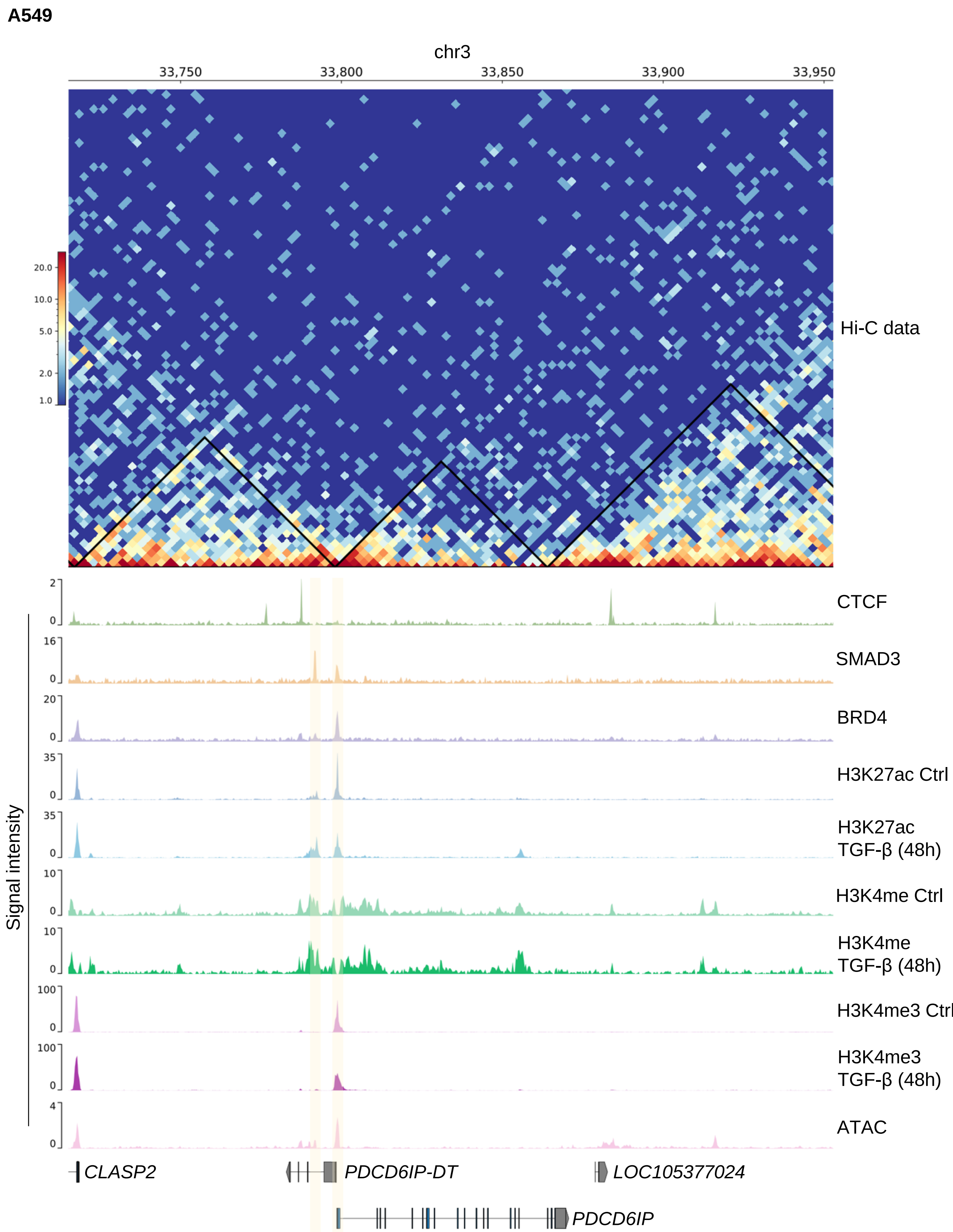
A549
chr3
33,750
33,800
33,850
33,900
33,950
20.0
10.0
5.0
2.0
1.0
Hi-C data
Signal intensity
CTCF
SMAD3
BRD4
H3K27ac Ctrl
H3K27ac TGF-β (48h)
H3K4me Ctrl
H3K4me TGF-β (48h)
H3K4me3 Ctrl
H3K4me3 TGF-β (48h)
ATAC
CLASP2
PDCD6IP-DT
LOC105377024
PDCD6IP

Suppl. Fig S3

Supplementary Figure S3. Genomic landscape of the *ALIX* locus in A549 cells. Hi-C 1D interaction frequency heatmap in A549 cells (top, 3 kbp resolution). Black triangles in the heatmap indicate topologically associating domains (TADs), which were predicted with a 3 kbp step. CTCF, SMAD3 (TGF-β-stimulated for 1.5 h), BRD4 ChIP-seq profiles in the genomic region surrounding the *ALIX* locus in A549 cells (GSE92782, GSE51510, GSE226487). ChIP-seq profiles of H3K27ac, H3K4me and H3K4me3 with or without TGF-β stimulation for 48 hours (CRA001325) are presented.

A549

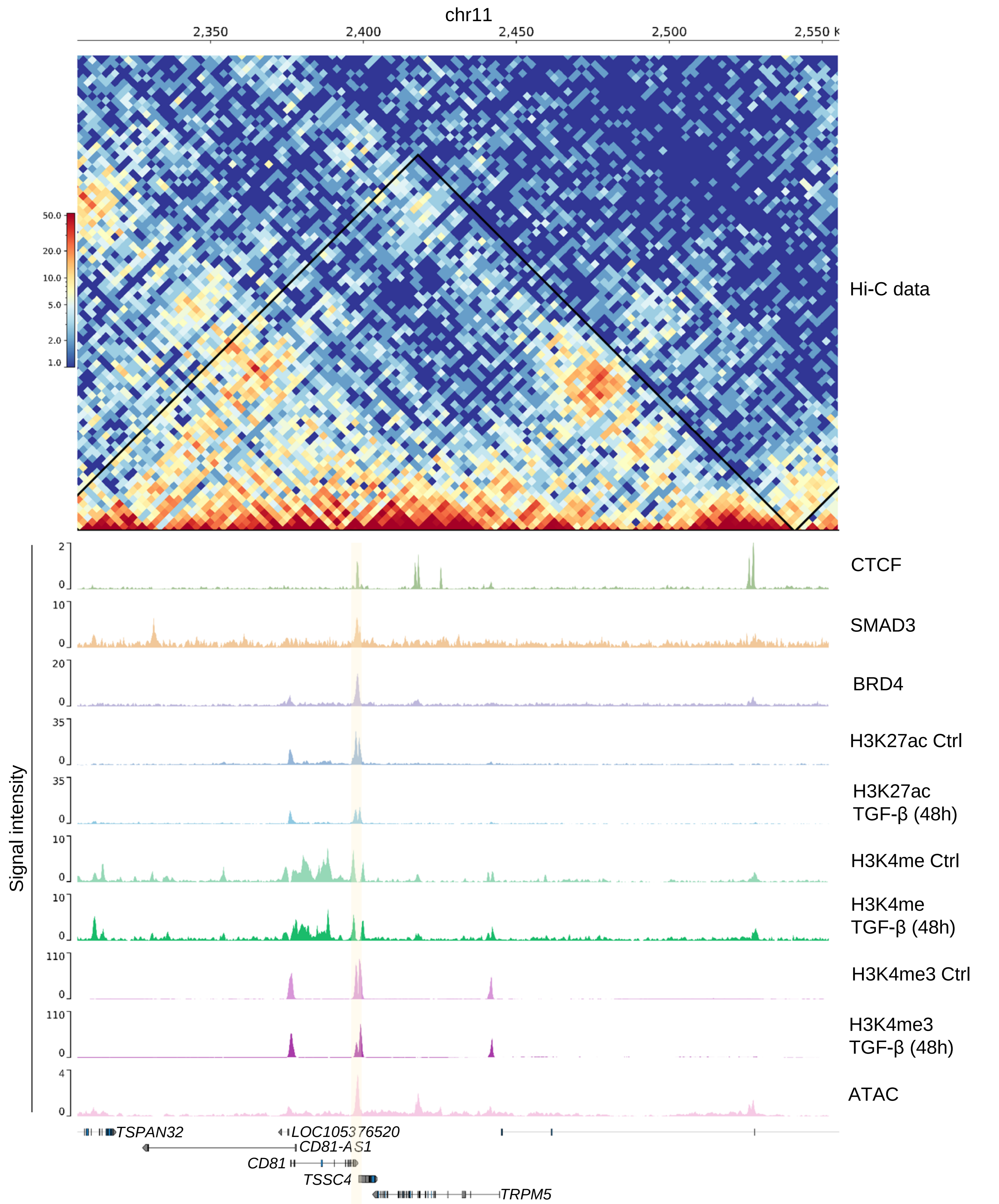

Suppl. Fig S4

Supplementary Figure S4. Genomic landscape of the *CD81* locus in A549 cells. Hi-C 1D interaction frequency heatmap in A549 cells (top, 3 kbp resolution). Black triangles in the heatmap indicate topologically associating domains (TADs), which were predicted with a 3 kbp step. CTCF, SMAD3 (TGF-β-stimulated for 1.5 h), BRD4 ChIP-seq profiles in the genomic region surrounding the *CD81* locus in A549 cells (GSE92782, GSE51510, GSE226487). ChIP-seq profiles of H3K27ac, H3K4me and H3K3me3 with or without TGF-β stimulation for 48 hours (CRA001325) are presented.

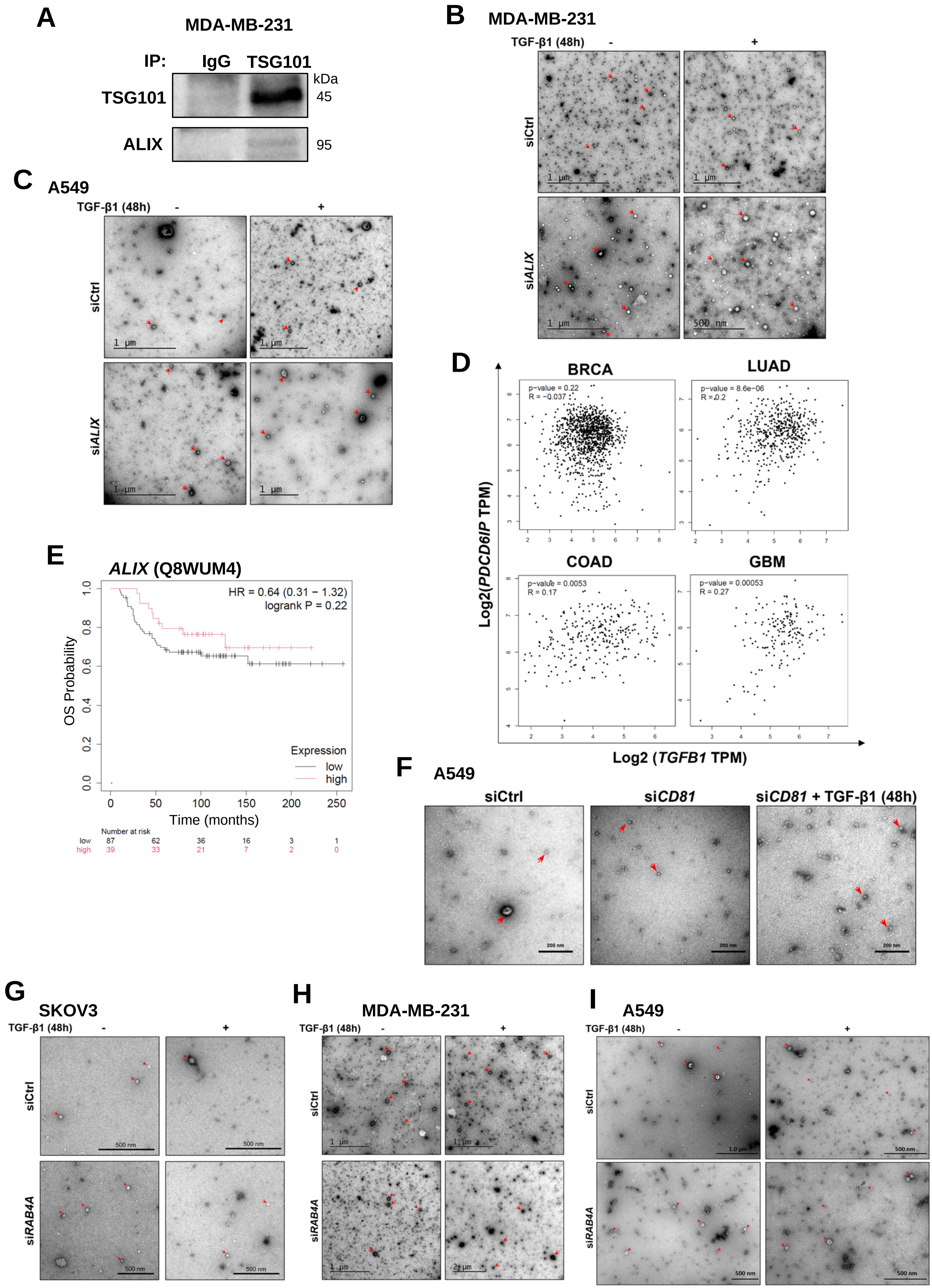
A
MDA-MB-231
IP: IgG TSG101
kDa
TSG101 45
ALIX 95
B
MDA-MB-231
TGF-β1 (48h) - +
siCtrl
siALIX
1 μm
500 nm
C
A549
TGF-β1 (48h) - +
siCtrl
siALIX
1 μm
D
BRCA
p-value = 0.22
R = -0.037
LUAD
p-value = 8.6e-06
R = 0.2
COAD
p-value = 0.0053
R = 0.17
GBM
p-value = 0.00053
R = 0.27
Log2(PDCD6IP TPM)
Log2 (TGFB1 TPM)
E
ALIX (Q8WUM4)
HR = 0.64 (0.31 − 1.32)
logrank P = 0.22
OS Probability
Expression
low
high
Time (months)
Number at risk
low 87 62 36 16 3 1
high 39 33 21 7 2 0
F
A549
siCtrl
siCD81
siCD81 + TGF-β1 (48h)
200 nm
G
SKOV3
TGF-β1 (48h) - +
siCtrl
siRAB4A
500 nm
H
MDA-MB-231
TGF-β1 (48h) - +
siCtrl
siRAB4A
1 μm
2 μm
I
A549
TGF-β1 (48h) - +
siCtrl
siRAB4A
1.0 μm
500 nm

Suppl. Fig S5

Supplementary Figure S5. Related to Figures 4, 5 and 7. TGF-β triggers EV release via an ALIX and CD81-independent pathway. (A) Protein complex formation between TSG101 and ALIX. MDA-MB-231 cell lysates were immunoprecipitated (IP) with TSG101-specific antibody or non-specific IgG, followed by SDS-PAGE and immunoblot with ALIX and TSG101 antibodies. Representative immunoblots of at least two independent biological replicates along with molecular mass markers in kDa are shown. **(B, C)** Representative TEM pictures of EVs isolated as VSF from MDA-MB-231 (B) and A549 (C) cells transiently transfected with control (siCtrl) or specific siRNA targeting *ALIX* (si*ALIX*) and stimulated with 5 ng/mL TGF-β1 or not (-) for 48 hours. Red arrows mark specific EVs; scale bars 500 nm or 1 µm, accordingly indicated in the figure. **(D)** Pearson correlation analysis of *PDCD6IP* (*ALIX*) mRNA expression in BRCA, LUAD, COAD and GBM relative to *TGFB1* mRNA expression, measured as transcripts per million (TPM) transformed by $\log_2$; data were obtained from TCGA. P- and R-values are listed. **(E)** ALIX protein levels did not correlate with overall survival (OS) outcome in human BRCA patients. BRCA samples were stratified and compared in the Kaplan-Meier plotter database using auto-select cut-off. **(F)** Representative TEM pictures of EVs isolated as VSF from A549 cells transiently transfected with control (siCtrl) or specific siRNA targeting CD81 (si*CD81*) and stimulated with 5 ng/mL TGF-β1 or not for 48 hours. Red arrows mark specific EVs. scale bars = 200 nm. **(G-I)** Representative TEM pictures of EVs isolated as VSF from SKOV3 (G), MDA-MB-231 (H) and A549 (I) cells transiently transfected with control (siCtrl) or specific siRNA targeting RAB4A (si*RAB4A*) and stimulated with 5 ng/mL TGF-β1 or not (-) for 48 hours. Red arrows mark specific EVs. Scale bars 500 nm, 1 or 2 µm, accordingly.
